# Developing a clinician-friendly rubric for assessing history-taking skills in medical undergraduates speaking English as a foreign language

**DOI:** 10.12688/mep.19911.1

**Published:** 2024-03-08

**Authors:** Takayuki Oshimi

**Affiliations:** 1 Office of Medical Education, School of Medicine, International University of Health and Welfare, Narita, Chiba, 286-8686, Japan

**Keywords:** history-taking skills, English for medical purposes, English as a foreign language (EFL), Occupational English Test (OET), rubric

## Abstract

**Background:**

The Occupational English Test (OET) is a globally recognized test for healthcare professionals who speak English as a foreign language (EFL). Although its speaking sub-test criteria are publicly accessible, effective application demands specialized assessor training. This study aimed to create a physician-friendly rubric for assessing the English history-taking skills of EFL medical undergraduates inspired by the OET speaking sub-test.

**Methods:**

Informed by the OET criteria, a rubric was tailor-made to assess the English history-taking skills of EFL medical undergraduates studying in Japan. Using this rubric, 14 physicians assessed the English history-taking skills of 134 sixth-year medical undergraduates. The study used exploratory factor analysis to ascertain its construct validity, evaluated the instrument’s reliability through Cronbach’s α and inter-rater reliability with chi-squared tests, and conducted a multiple regression analysis, ensuring adherence to key regression assumptions.

**Results:**

A total of three key factors were found: linguistic-clinical distinction, communication dynamics, and medical comprehension. The rubric’s internal consistency was verified, achieving a Cronbach’s α of 0.799. Discrepancies in assessor scores highlighted the need for calibration. In total, four criteria emerged as vital in assessing the students’ performance.

**Conclusions:**

The tailored rubric effectively assesses the English history-taking skills of EFL medical undergraduates.

## Introduction

The practice of medicine deeply hinges on effective communication. History-taking, characterized by extracting pertinent personal, psychosocial, and symptomatic data from a patient, is central to this process ( Keifenheim *et al.*, 2015). Through proficient history-taking, physicians can obtain approximately 60% to 80% of the crucial information necessary for an accurate diagnosis ( Hampton *et al.*, 1975; Kassirer, 1983; Peterson *et al.*, 1992; Roshan & Rao, 2000; Sandler, 1980). This method ensures diagnostic precision and enhances patient and physician satisfaction, ultimately leading to optimal health outcomes ( Fortin *et al.*, 2002; Hatem *et al.*, 2007; Novack *et al.*, 1992; Sanson-Fisher & Maguire, 1980; Windish *et al.*, 2005).

English is predominantly used in international medical conferences, journals, and collaborative research endeavors ( Chan & Graham, 2011; Jenkins, 2009; Warde *et al.*, 2018). The linguistic challenges inherent in navigating this terrain are amplified for medical undergraduates for whom English is a foreign language (EFL). For these medical undergraduates whose first language is not English, the requirement to express themselves in English can diminish their confidence, especially during history-taking ( Mirza & Hashim, 2010), culminating in potential misunderstandings that jeopardize patient trust and affect diagnostic clarity.

In regions where English is used as an official language in healthcare settings, standardized tests like the Occupational English Test (OET), developed by Professor Tim McNamara at the University of Melbourne, serve as tools to assess these indispensable communication skills among healthcare professionals who received their training in countries where languages other than English are used ( Mladenovic *et al.*, 2023).

Prior to taking the big step of pursuing a healthcare career in an English-speaking country, many EFL medical undergraduates would benefit from having their communication skills tested using a bespoke assessment mechanism. The OET speaking sub-test assessment criteria, which have been rigorously tested and validated through empirical research ( Elder *et al.*, 2013; Frost *et al.*, 2015; Frost *et al.*, 2016; Pill & Knoch, 2014; Séguis & McElwee, 2019), serve as a promising starting point. This publicly accessible assessment tool consists of two main domains: Linguistic Criteria and Clinical Communication Criteria (see extended data for details; Oshimi, 2024). However, to employ the OET more extensively, assessors must receive specific training tailored to the OET’s assessment context ( O’Hagan *et al.*, 2015). While such specialized training is apt for decisions concerning hiring healthcare professionals, its application seems too intensive for preliminary evaluations of EFL medical students in contexts such as formative assessments to refine their English history-taking skills.

This research endeavors to fill this gap and surmount this challenge. Drawing inspiration from the foundational criteria of the OET speaking sub-test and assimilating insights from clinical communication experts in English-speaking countries, the objective is to devise stringent yet equitable criteria for assessing the English history-taking proficiency of EFL medical undergraduates. Particularly crucial in this pursuit is the creation of an assessment tool tailored for clinicians tasked with teaching history-taking skills in English while ensuring that it places minimal linguistic demands on assessors.

In essence, this research aims to devise a physician-friendly rubric inspired by the OET speaking sub-test to validly and reliably assess the English history-taking capabilities of EFL medical undergraduates.

## Methods

### Ethical considerations and participant consent

The research was conducted under International University of Health and Welfare (IUHW) Ethics Committee Approval Number 16-Io-246, dated April 5 ^th^, 2017. The research ethics committee endorsed access to the assessment data, and informed consent was obtained in written form from all 134 study participants.

### Origins of the assessment framework

This rubric’s conception traces its foundation to the OET speaking sub-test (see Appendix 1 in the extended data for details), a recognized and influential benchmark in assessing English proficiency within a clinical setting ( Elder *et al.*, 2013; Frost *et al.*, 2015; Frost *et al.*, 2016; Pill & Knoch, 2014; Séguis & McElwee, 2019). The specific points of the OET assessment framework are summarized below to illustrate the genesis of the newly proposed rubric.

***Linguistic criteria***

All components utilize a 0–6 scale.

- **Intelligibility**: Measures speech clarity, including pronunciation and prosodic features. A score of 6 denotes impeccable pronunciation and prosody, while lower scores reflect increasing L1 accent influence, with 0 being non-responsive.
- **Fluency**: Assesses speech flow and tempo. A score of 6 indicates seamless speech, whereas scores closer to 0 reflect disruptions such as frequent pauses or repetitions.
- **Appropriateness of language**: Assesses appropriate register, tone, and vocabulary use. Perfect appropriateness earns a 6, with decreasing scores indicating increasing inappropriateness.
- **Resources of grammar and expression**: A high score signifies mastery over a diverse range of grammar and vocabulary patterns, while lower scores point to limited vocabulary or grammatical errors.

***Clinical communication criteria***

All components utilize a 0–3 scale, with 0 defined as ineffective and 3 as adept.

- **Relationship building**: Measures skills in initiating interactions, displaying empathy, and understanding the patient's emotions, including aspects like appropriate greetings, and showing respect.
- **Understanding and incorporating the patient's perspective**: Assesses the clinician's ability to capture and address the patient's concerns, including effective explanation and cue recognition.
- **Providing structure**: Reviews the organization and flow of the interview, emphasizing sequence, topic signposting, and structuring techniques.
- **Information gathering**: Assesses skills in collecting and clarifying patient data, emphasizing active listening, open-ended questions, and summarization.
- **Information giving**: Analyzes proficiency in conveying information to patients, with emphasis on assessing patient knowledge, ensuring comprehension, and inviting feedback.

Drawing from these comprehensive parameters of the OET, the present research aims to design a robust assessment tool tailored to the needs of EFL medical undergraduates for history-taking tasks.

### History-taking-centric rubric adaptation

The task of refining a rubric designed to assess the history-taking abilities of EFL medical undergraduates was carried out based on the collective expertise of four in-house faculty members. All four faculty members, EFL physicians, have specialized in teaching medical communication skills in English in Japan. Their combined work experience, ranging from two to 15 years, added depth and breadth to the rubric’s validation process. To ensure a comprehensive and relevant rubric, the faculty members actively engaged in iterative discussions, sharing their experiences and insights to refine the assessment tool. Their contributions were synergized through interactive sessions, allowing for seamless linguistic and clinical expertise integration.

The adaptation process aimed to make the criteria more understandable and applicable to clinicians. The following points showcase the changes from the OET’s original structure to the LCM Rubric Version 1. Major adaptations include:

- **Linguistic criteria 1–4 (L1–4)**: Streamlined descriptors for enhanced clarity.
- **Clinical communication criteria 1–4 (C1–4)**: Changed from item selection to a checklist format to focus on objectivity and clarity.
- **Medical interview criteria 1–3 (M1–3)**: Incorporated strategic elements such as patient safety, medical history components, and comprehensiveness of history of present illness.
- **Global rating 1–2 (G1–2)**: Introduced a comprehensive assessment comparing students’ abilities to those of their counterparts in English-speaking countries.

Patient safety is consistently featured in objective structured clinical examination (OSCE) checklists, underscoring its importance. The emphasis on the history of present illness is derived from its critical role in generating differential diagnoses and guiding medical decision-making. This significance is buttressed by Skeff’s findings, asserting that the history of present illness (HPI) is pivotal for an in-depth analysis of a patient’s condition ( Skeff, 2014). The Global Rating category provides a comprehensive assessment of a student’s skills in comparison to their counterparts in English-speaking countries.

The adapted rubric focused on history-taking is termed the **LCM Rubric Version 1**. The acronym " **LCM**" denotes the three pillars of the rubric: **L**inguistic, **C**linical Communication, and **M**edical Interview. Details are provided in **Appendix 2 of the extended data** ( Oshimi, 2024).

### Assessment

In July 2022, all 134 sixth-year medical undergraduates at the International University of Health and Welfare (IUHW) in Japan participated in an assessment of their English history-taking skills, which was also an integral component of their mandatory post-clinical-clerkship objective structured clinical examination (post-CC OSCE). No minors were included in this assessment.

During the first two years of their medical education, these students were extensively trained in medical subjects taught in English. This foundation was bolstered by a 120-hour medical English course tailored to enhance their English history-taking proficiency. As an ongoing measure of their English language proficiency, they had sat the Test of English as a Foreign Language Institutional Testing Program (TOEFL ITP) seven times before their post-CC OSCE assessment.

The 12-minute English history-taking assessment was structured around a simulated patient setting to replicate a realistic medical scenario. A total of eight individuals acted as simulated patients: six were IUHW faculty members, and two were volunteers, including a single native English speaker. The inter-simulated patient reliability was also statistically assessed regarding the potential bias introduced by faculty members acting as simulated patients. Although using only one native English speaker might be perceived as a limitation, given the emphasis on English history-taking skills, this composition was chosen to replicate a diverse patient base that mirrors more closely the non-Japanese patient population residing in Japan who are likely to seek medical care. Each simulated patient received individualized training, lasting one hour, to consistently play the role of a patient suffering from a headache, ultimately diagnosed as a migraine (see **Appendix 3** of the extended data for details of the patient prompts; Oshimi, 2024).

To comprehensively document each student’s performance, each simulated consultation room was fitted with two ceiling-embedded video cameras, capturing both the student and the simulated patient from dual angles. The video recordings of satisfactory visual and audio quality were the primary means for assessors to gauge the students’ performance.

The IUHW School of Medicine curated a group of 14 assessors from its broad professional connections. These assessors possess clinical education backgrounds in English-speaking countries. They were remunerated for their involvement in the assessment as external, objective third parties. The researcher did not have access to the full, personal details regarding these assessors and simulated patients and was thus unable to contact them individually after they had finished the work they had been contracted to do. As a result, authorization to access the post-CC OSCE scores of the students for the purpose of conducting this research had to be obtained from the institutional ethics committee.

Their assessments, primarily centered on the Global Rating of the LCM Rubric Version 1, were the principal metric of a student’s abilities. This Global Rating consists of two parts:

- **Global rating 1 (G1)**: Assessors checked if students engaged in clinically appropriate behaviors such as patient-centered interviewing, respect towards the patient, rapport-building, and professional conduct during the medical interview.
- **Global rating 2 (G2)**: Assessors rated students based on predefined levels of expertise, ranging from the level of an outstanding student who has finished their core clinical clerkships to that of a novice who has yet to begin their clinical clerkship.

To facilitate the assessment process, each pair of assessors reviewed the performances of 19 to 20 students using video recordings accessible on a cloud platform. The LCM Version 1 was provided via Google Forms to ensure a consistent assessment approach. This rubric, familiar to the students beforehand, highlighted the essential Medical Interview Components they were expected to cover. Furthermore, assessors were directed to consult an external resource ( Benchmark Education Solutions, 2023) for an in-depth understanding of the linguistic and clinical communication criteria embedded within the rubric.

### Data analysis

***Construct validity***

Exploratory factor analysis (EFA): The dataset comprised 13 items from the rubric, complemented by 12 variables derived from TOEFL ITP scores. These TOEFL ITP scores provided a longitudinal view of English language proficiency as measured on three separate occasions: scores taken on admission, scores acquired after completing the initial 2-year medical education in English, and scores obtained before the history-taking assessment. Students were asked to sit the TOEFL ITP test on seven occasions during their medical education, twice in the first year and once yearly up to their sixth year. Each test result comprised four items detailing the total, Section 1 (Listening), Section 2 (Grammar), and Section 3 (Reading) scores. Consequently, an EFA was performed on these 25 aggregate variables. The Kaiser criterion guided the identification of distinct factors, and the analysis incorporated a Promax rotation. It is noteworthy that, given the non-obligatory nature of the TOEFL ITP exams after the third year of medical education, there were occurrences of missing data. When the data from the seventh TOEFL ITP test was unavailable, scores from the sixth or fifth test were pragmatically utilized as substitutes. This adaptive approach was made necessary by the elective status of the TOEFL ITP following the third year of their study.

***Reliability***

Internal consistency: Cronbach’s α coefficients were determined to verify the internal consistency of the rubric factors and the TOELF ITP scores.

Inter-rater reliability: Variability among assessors covering the new assessment criteria was appraised using chi-squared tests.

***Multiple regression analysis***

A multiple regression analysis designated the Global Rating 2 (G2) as the dependent variable. The independent variables encompass the rubric scores from criteria other than the Global Rating and specific TOEFL ITP scores: scores taken on admission, scores acquired after completing English education, and scores obtained before the history-taking assessment. Throughout this analysis, attention was accorded to regression assumptions, including linearity, multicollinearity, and homoscedasticity.

***Significance threshold***

All statistical tests were anchored to a significance level set at *P* =0.05 (two-tailed). *P*-values below this threshold were interpreted as indicative of statistical significance. Statistical tests were conducted with SPSS Statistics 26 (IBM Corporation, Armonk, NY, USA).

## Results

### Scores on LCM Rubric Version 1

**Figure 1.**
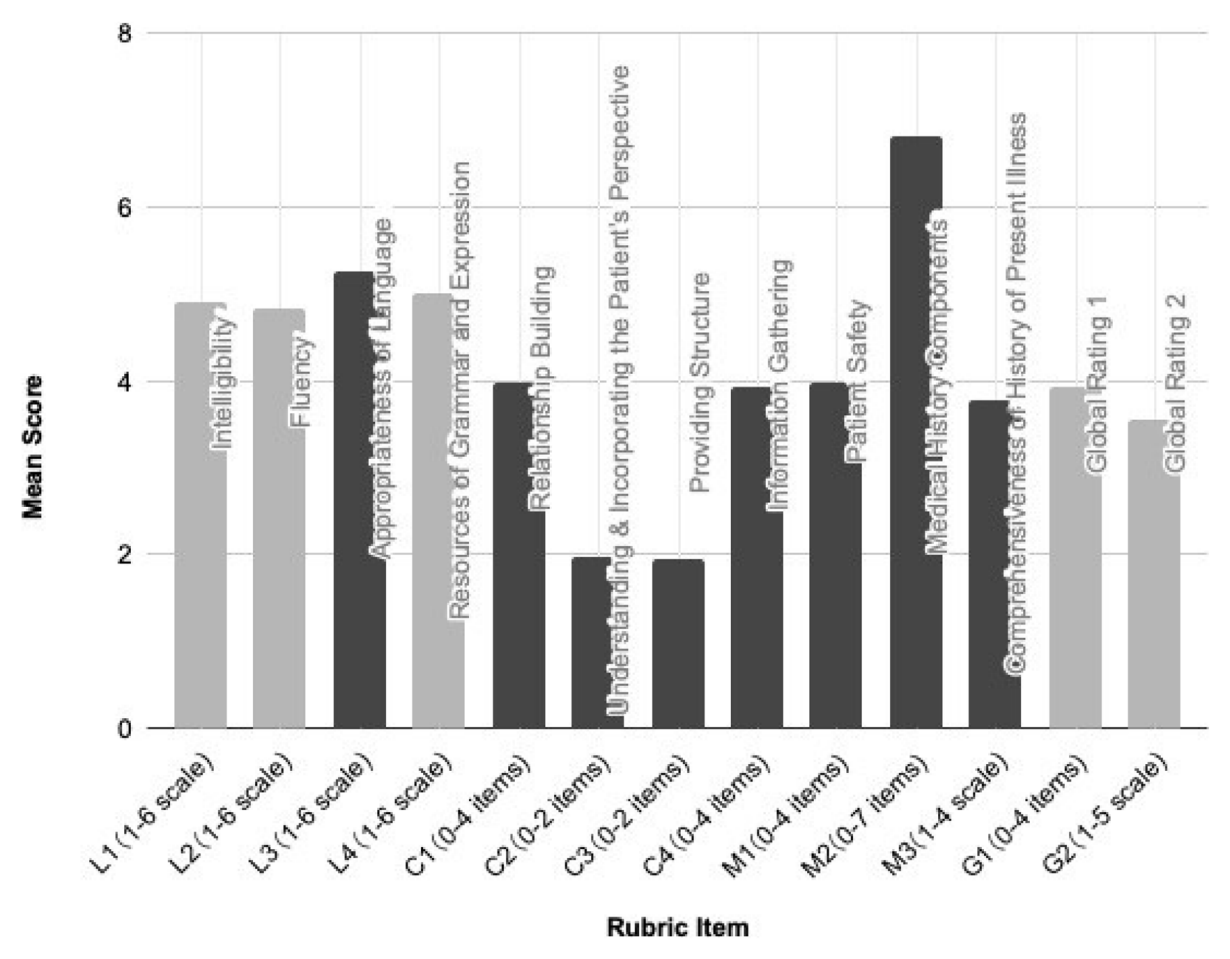
Scores on LCM Rubric Version 1. N=134 students; assessed by two assessors each.

Figure 1 presents the mean scores on the LCM Rubric Version 1.

A notable ceiling effect was discerned for eight items: L3, C1, C2, C3, C4, M1, M2, and M3. The prominence of this effect might stem from the rubric being revealed to examinees before the assessment, serving as an instrument guiding their preparations. Despite the absence of a floor effect, its educational implications were positive: the rubric, serving as a preparatory guideline, likely influenced the high-scoring trend.

***Construct validity***

The construct validity of the LCM Version 1 rubric was confirmed with EFA. Initial analysis of all 25 variables encountered computational challenges, notably from the C1 variable (Relationship Building) due to an influential outlier. Additionally, the interdependence of comprehensive and skill-specific TOEFL scales, influenced by the TOEFL scoring structure, presented challenges.

In total, four significant factors emerged when EFA was refined to 15 variables, yielding a Cronbach’s alpha coefficient of 0.690, hinting at potential variances introduced by the TOEFL ITP scores. Table 1 shows the factor loadings for each of the 12 variables against the three identified factors.

**Table 1.** Refined EFA variables and identified factors. *N*=134 students; assessed by two assessors each.

Rubric item	Identified factors		
	Factor 1	Factor 2	Factor 3
	Linguistic-clinical distinction	Communication dynamics	Medical comprehension
**L1 (1-6 scale)**	**0.882**	-0.116	0.06
**L2 (1-6 scale)**	**0.917**	-0.054	0.05
**L3 (1-6 scale)**	**0.689**	0.018	-0.157
**L4 (1-6 scale)**	**0.839**	-0.08	-0.048
**C2 (0-2 items)**	0.186	-0.019	-0.227
**C3 (0-2 items)**	-0.093	0.349	0.353
**C4 (0-4 items)**	-0.03	**0.72**	0.053
**M1 (0-4 items)**	-0.147	0.299	0.07
**M2 (0-7 items)**	-0.005	-0.083	**0.427**
**M3 (1-4 scale)**	0.107	0.066	**0.605**
**G1 (0-4 items)**	0.088	**0.742**	-0.24
**G2 (1-5 scale)**	**0.503**	0.342	0.1
**L1**: Intelligibility **L2**: Fluency **L3**: Appropriateness of Language **L4**: Resource of Grammar and Expression		**C1**: Relationship Building **C2**: Patient's Perspective **C3**: Providing Structure **C4**: Information Gathering	
**M1**: Patient Safety **M2**: Medical History Components **M3**: Comprehensiveness of HPI		**G1**: Quality **G2**: Overall Performance	
Extraction Method: Maximum Likelihood Rotation Method: Promax with Kaiser Normalization Note: Rotation converged in 4 iterations			

The three factors identified in the EFA are described as follows: Factor 1, labeled “ **Linguistic-Clinical Distinction**,” differentiates between linguistic aspects (L) and clinical components (C & M). Factor 2, labeled “ **Communication Dynamics**,” encompasses elements that foster doctor-patient rapport-building (G1), facilitate information gathering (C4), and include other relevant linguistic and medical tasks (L & M). Thirdly, Factor 3, labeled “ **Medical Comprehension**,” highlights the subtleties in medical history components (M2), the comprehensiveness of the history of the present illness (M3), and the interpretation of patient perspectives (C2).

The divergence between rubric items and TOEFL scores suggested an iterative EFA approach. Without TOEFL scores, the Cronbach’s alpha improved to 0.799. However, caution is advised due to skews in items like Relationship Building (C1) and potential ceiling effects in other variables.

In summary, while the EFA supports the construct validity of the LCM Rubric Version 1, nuances related to specific variables and consistency are notable. Nevertheless, the LCM Rubric Version 1 stands as a promising assessment tool.

### Reliability

***Internal consistency***

Excluding the TOEFL ITP scores and the skewed variable C1, the rubric items achieved a Cronbach’s α coefficient of 0.799, indicating satisfactory reliability. Table 2 details Cronbach’s α values for individual variables.

**Table 2.** Cronbach’s α coefficient summary. *N*=134 students; assessed by two assessors each.

Rubric item	Scale mean value	Scale variance	Item-total correlation	Squared multiple correlation	Cronbach's α
**L1 (1-6 scale)**	45.08	11.322	0.744	0.668	**0.746**
**L2 (1-6 scale)**	45.14	10.866	0.805	0.736	**0.736**
**L3 (1-6 scale)**	44.72	12.179	0.571	0.432	**0.772**
**L4 (1-6 scale)**	44.97	12.456	0.677	0.592	**0.758**
**C2 (0-2 items)**	48.01	16.207	0.084	0.062	**0.805**
**C3 (0-2 items)**	48.04	15.877	0.222	0.194	**0.801**
**C4 (0-4 items)**	46.05	15.47	0.327	0.372	**0.795**
**M1 (0-4 items)**	46.01	16.281	0.028	0.091	**0.806**
**M2 (0-7 items)**	43.19	15.616	0.127	0.139	**0.808**
**M3 (1-4 scale)**	46.21	14.753	0.377	0.254	**0.79**
**G1 (0-4 items)**	46.05	15.622	0.329	0.366	**0.796**
**G2 (1-5 scale)**	46.39	11.043	0.681	0.519	**0.757**
**L1**: Intelligibility **L2**: Fluency **L3**: Appropriateness of Language **L4**: Resource of Grammar and Expression			**C1**: Relationship Building **C2**: Patient's Perspective **C3**: Providing Structure **C4**: Information Gathering		
**M1**: Patient Safety **M2**: Medical History Components **M3**: Comprehensiveness of HPI			**G1**: Quality **G2**: Overall Performance		

While the α coefficient’s stability persisted with each variable’s exclusion, emphasizing the rubric’s integrity, its inclusion of TOEFL ITP scores reduced the coefficient to 0.690. Variables like C1 showed significant skewness, and others, including L3, C1 to C4, and M1 to M3, presented ceiling effects, warranting careful interpretation in subsequent analyses.

***Inter-rater reliability***

Rating consistency across assessors was examined for the newly added criteria: M1, M2, M3, G1, and G2. With the chi-squared test due to the categorical score data, significant variations were observed in all criteria. Specifically, two raters assessing the same 19 students showed marked differences only in the M1 criterion ( *P* =0.046). This emphasizes the variability in scoring and the importance of thorough training for consistent evaluations. Additionally, inter-simulated patient reliability mirrored the procedure for inter-rater reliability, revealing no significant differences among simulated patients.

***Multiple regression analysis***

In the regression analysis, the C1 data showed significant skewness, predominantly scoring 4 with one score of 2. Given its pronounced skewness, C1 was omitted, and no transformation was applied as it would be ineffective. The regression model had a strong correlation with a multiple correlation coefficient (R) of 0.686, and a coefficient of determination (R ^2^) of 0.471, being statistically significant ( *P* < 0.001). The F-statistic was 16.633 with degrees of freedom (df) of 13 for regression, and 243 for residuals, and the model's standard error was 0.728 ( Table 3).

**Table 3.** Multiple regression analysis results for the relationship between specific criteria and Global Rating 2 (G2) scale. *N* =134 students; assessed by two assessors each.

Predictor	B (Unstandardized coefficient)	Standard error	Beta (Standardized coefficient)	t-value	p-value	95% Confidence interval (B)	Tolerance	VIF
**Intercept** **(Constant)**	-7.178	1.796	-	-3.996	0	(-10.716, -3.640)	-	-
**L1 (1-6** **scale)**	0.103	0.093	0.09	1.113	0.267	(-0.079, 0.285)	0.33	3.031
**L2 (1-6** **scale)**	0.126	0.101	0.113	1.246	0.214	(-0.073, 0.324)	0.265	3.773
**L3 (1-6** **scale)**	0.241	0.068	0.215	3.561	**0**	(0.108, 0.374)	0.598	1.671
**L4 (1-6** **scale)**	0.287	0.099	0.211	2.914	**0.004**	(0.093, 0.482)	0.415	2.409
**C2 (0-2** **items)**	0.147	0.322	0.023	0.458	0.647	(-0.486, 0.781)	0.885	1.13
**C3 (0-2** **items)**	0.372	0.21	0.091	1.77	0.078	(-0.042, 0.785)	0.822	1.217
**C4 (0-4** **items)**	0.581	0.17	0.175	3.413	**0.001**	(0.245, 0.916)	0.826	1.21
**M1 (0-4** **items)**	0.676	0.35	0.094	1.932	0.054	(-0.013, 1.365)	0.918	1.089
**M2 (0-7** **items)**	-0.033	0.105	-0.016	-0.318	0.751	(-0.239, 0.173)	0.844	1.185
**M3 (1-4** **scale)**	0.306	0.111	0.147	2.742	**0.007**	(0.086, 0.525)	0.761	1.314
**Admission** **TOEFL ITP**	-0.002	0.002	-0.078	-0.709	0.479	(-0.006, 0.003)	0.179	5.584
**Post-English** **Ed. TOEFL** **ITP**	0.002	0.002	0.103	0.916	0.361	(-0.002, 0.006)	0.174	5.764
**Pre-History-** **taking** **TOEFL ITP**	0	0.002	-0.026	-0.241	0.81	(-0.004, 0.003)	0.184	5.446
**L1**: Intelligibility **L2**: Fluency **L3**: Appropriateness of Language **L4**: Resource of Grammar and Expression				**C1**: Relationship Building **C2**: Patient's Perspective **C3**: Providing Structure **C4**: Information Gathering				
**M1**: Patient Safety **M2**: Medical History Components **M3**: Comprehensiveness of HPI				**G1**: Quality **G2**: Overall Performance				

In total, four specific criteria emerged with a statistically significant relationship with the dependent variable Global Rating 2 (G2), which assesses students’ clinical competency progression through core clinical clerkships such as appropriateness of language (L3), resources of grammar and expression (L4), information gathering (C4), comprehensiveness of history of present illness (M3).

Despite their incorporation into the model, TOEFL ITP scores did not show a statistically significant relationship with the dependent variable. The finding that TOEFL ITP scores did not fall below the significance threshold underscores the idea that the rubric assesses skills distinct from those gauged by a standard English language proficiency test like the TOEFL ITP.

## Discussion

This research aimed to create a specialized rubric to assess the English history-taking skills of EFL medical undergraduates. Drawing inspiration from the OET speaking sub-test, the resultant LCM Rubric Version 1 was formulated and validated through a comprehensive empirical study.

Upon evaluating the mean scores across the various LCM Rubric Version 1 criteria, a pronounced ceiling effect was evident for eight items. This effect might be attributed to several factors. One potential reason could be the prior exposure of examinees to the rubric, which could have facilitated their preparation. This observation suggests a dual role of the rubric: it acts not only as an assessment tool but also as a guiding instrument in student preparation. This alignment with the rubric might indicate that the majority of students were able to execute on examination what they were emphasized during their education. Such an interpretation suggests that the educational outcomes aimed for were effectively achieved. From this perspective, the ceiling effect might not necessarily be viewed negatively, but rather as an indication that a large proportion of students successfully met or even exceeded the standards set, reflecting positively on the educational approach. However, while this data concentration towards the upper limit can be seen as a testament to the efficacy of the educational emphasis, it also prompts reflections on the potential leniency of assessors or the possible influence of preparation on elevating scores.

Construct validity, assessed through exploratory factor analysis, revealed three distinguishable factors: linguistic-clinical distinction, communication dynamics, and medical comprehension. The highlighted factors emphasize the importance of differentiating linguistic proficiency from clinical communication skills, reinforcing the value of specialized instruments like the LCM Rubric for assessing occupation-specific skills in EFL medical undergraduates. It is worth noting that the inclusion of the TOEFL ITP scores had implications on internal consistency, as seen with the sub-threshold Cronbach’s alpha value of 0.690. This finding highlights the rubric’s distinctiveness against general English proficiency tests and validates its tailored approach toward assessing specialized communication skills.

While the rubric’s internal consistency showed commendable robustness with a Cronbach’s α coefficient of 0.799, certain items posed challenges, notably the C1 variable, which showed significant skewness. The discrepancies between assessor scores accentuate the necessity of rigorous assessor training. This finding will ensure assessment consistency and reduce variability stemming from subjective rubric interpretations.

The multiple regression analysis underscored four vital criteria directly associated with the Global Rating 2 (G2) assessment of clinical competency. Among these, the comprehensiveness of history of present illness (M3) stood out with marked significance, emphasizing the foundational importance of HPI in clinical practice. While it is common for novice medical students to lean on standardized expressions, the depth and intricacy of HPI must be considered. Hence, EFL undergraduate curricula in medical English communication should prioritize an exhaustive exploration of HPI. Moreover, while other criteria like appropriateness of language (L3) and resources of grammar and expression (L4) also have a bearing on performance, it is noteworthy that the LCM Rubric’s assessment method is markedly different from that of general English proficiency tests, as evidenced by the non-significant relationship with TOEFL ITP scores.

In summary, the LCM Rubric Version 1, informed by the OET speaking sub-test criteria, provides a tailored approach for assessing the English history-taking skills of EFL medical undergraduates. The validation and findings from this study suggest its potential utility in educational settings, bearing implications for curriculum development and student training in global medical communication.

### Limitations

The study examining English history-taking abilities of EFL medical undergraduates at a private Japanese medical school has limitations. The unique emphasis on English at IUHW may limit the findings' generalizability to other academic settings. Given its primary focus on Japan, it is important to consider linguistic nuances in different international medical contexts.

Relying only on the OET speaking sub-test might omit some facets of students' linguistic proficiency. The selection of assessors, despite their diversity, introduces potential bias, and limited data on them could affect the study's depth. The specific focus on migraines and a particular cohort may not capture the broader EFL medical experience. Video recordings, while practical, might only partially capture face-to-face interactions. Questions about sample representativeness and the influence of access to post-CC OSCE scores on the study’s objectivity are worth noting.

### Recommendations for further research

This study underscores the importance of expansive inter-institutional collaboration between medical schools in Japan and their international counterparts to achieve a richer, more diverse participant base. Integrating artificial intelligence with the current rubric could optimize assessments in terms of efficiency and cost. Feedback on the LCM Rubric Version 2 from the assessors will further inform assessment refinement. Although this research offers valuable insights into EFL medical undergraduates’ English history-taking abilities at IUHW, the highlighted limitations set the direction for future studies. Broader collaborations, technological integration, and assessor feedback will refine assessment approaches.

## Data Availability

Given the sensitive nature of the data, especially considering that it pertains to student grades and the involvement of external assessors, the institutional ethics committee of the International University of Health and Welfare (IUHW) mandated stringent controls over data access. In alignment with our commitment to ensuring the confidentiality and protection of participant data, particularly the post-CC OSCE grades, the raw data from this study is not publicly accessible. Participants provided written consent to use their grades strictly for research purposes, not including public dissemination. This approach fully complies with the data protection concerns highlighted by the Institutional Review Board (IRB) at IUHW, which emphasizes stringent measures for data privacy and ethical handling of sensitive information. To maintain the integrity of the research and honor our confidentiality commitments, we have established a structured process for accessing the data under specific, ethically approved conditions, as also reviewed and sanctioned by the IUHW Research Ethics Committee. The process is as follows: Researchers interested in accessing the data should initially contact the corresponding author at oshimi@iuhw.ac.jp with a comprehensive explanation of the data's intended use. Subsequently, an official request must be submitted to the IUHW Research Ethics Committee. This request should articulate a transparent and ethical purpose for data usage, aligning with the study's objectives and the committee's ethical standards. Conditional access to the data may be granted based on the committee's review, ensuring that the request strictly adheres to the ethical guidelines and the study's objectives. This structured approach underscores our adherence to the guidelines stipulated by the IRB. It demonstrates our unwavering commitment to upholding data privacy while facilitating ethically sound research that aligns with the study's purpose. Appendix 1 (SPEAKING Assessment Criteria and Level Descriptors) is publicly available by the OET. For access to the full document, please visit the provided URL: https://prod-wp-content.occupationalenglishtest.org/resources/uploads/2018/08/22102547/speaking-assessment-criteria-updated-2018.pdf. Zenodo: Developing a clinician-friendly rubric for assessing history-taking skills in medical undergraduates speaking English as a foreign language. https://zenodo.org/records/10610376 ( Oshimi, 2024). This project contains the following extended data: Appendix 2.pdf (LCM Rubric Version 1) Appendix 3.pdf (Patient Prompts) Data are available under the terms of the Creative Commons Attribution 4.0 International license (CC-BY 4.0).
