# Developing a clinician-friendly rubric for assessing history-taking skills in medical undergraduates speaking English as a foreign language

**DOI:** 10.12688/mep.19911.3

**Published:** 2025-08-28

**Authors:** Takayuki Oshimi

**Affiliations:** 1 Office of Medical Education, School of Medicine, International University of Health and Welfare, Narita, Chiba, 286-8686, Japan

**Keywords:** history-taking skills, English for medical purposes, English as a foreign language (EFL), Occupational English Test (OET), rubric

## Abstract

**Background:**

ChatGPT is a large language model that uses deep learning techniques to generate human-like texts. ChatGPT has the potential to revolutionize medical education as it acts as an interactive virtual tutor and personalized learning assistant. We assessed the use of ChatGPT and other Artificial Intelligence (AI) tools among medical faculty in Uganda.

**Methods:**

We conducted a descriptive cross-sectional study among medical faculty at four public universities in Uganda from November to December 2023. Participants were recruited consecutively. We used a semi-structured questionnaire to collect data on participants’ sociodemographics and the use of AI tools such as ChatGPT. Our outcome variable was the use of ChatGPT and other AI tools. Data were analyzed in Stata version 17.0.

**Results:**

We recruited 224 medical faculty, majority [75% (167/224)] were male. The median age (interquartile range) was 41 years (34–50). Almost all medical faculty [90% (202/224)] had ever heard of AI tools such as ChatGPT. Over 63% (120/224) of faculty had ever used AI tools. The most commonly used AI tools were ChatGPT (56.3%) and Quill Bot (7.1%). Fifty-six faculty use AI tools for research writing, 37 for summarizing information, 28 for proofreading work, and 28 for setting exams or assignments. Forty faculty use AI tools for nonacademic purposes like recreation and learning new skills. Faculty older than 50 years were 40% less likely to use AI tools compared to those aged 24 to 35 years (Adjusted Prevalence Ratio (aPR):0.60; 95% Confidence Interval (CI): [0.45, 0.80]).

**Conclusions:**

The use of ChatGPT and other AI tools was high among medical faculty in Uganda. Older faculty (>50 years) were less likely to use AI tools compared to younger faculty. Training on AI use in education, formal policies, and guidelines are needed to adequately prepare medical faculty for the integration of AI in medical education.

## Introduction

The practice of medicine deeply hinges on effective communication. History-taking, which involves extracting pertinent personal, psychosocial, and symptomatic data from a patient, is central to this process ( Keifenheim *et al.*, 2015). Through proficient history-taking, physicians can gather approximately 60% to 80% of the essential information needed for an accurate diagnoses ( Hampton *et al.*, 1975; Kassirer, 1983; Peterson *et al.*, 1992; Roshan & Rao, 2000; Sandler, 1980). This approach not only ensures diagnostic accuracy but also boosts both patient and physician satisfaction, ultimately leading to better health outcomes ( Fortin *et al.*, 2002; Hatem *et al.*, 2007; Novack *et al.*, 1992; Sanson-Fisher & Maguire, 1980; Windish *et al.*, 2005).

English dominates international medical conferences, journals, and collaborative research endeavors ( Chan & Graham, 2011; Jenkins, 2009; Warde *et al.*, 2018). The linguistic challenges in navigating this environment are significant for medical undergraduates who speak English as a foreign language (EFL). For these students, communicating in English can reduce their confidence, especially during history-taking ( Mirza & Hashim, 2010), potentially leading to misunderstandings that compromise patient trust and affect diagnostic clarity.

In regions where English is the official language in healthcare, standardized tests like the Occupational English Test (OET), developed by Professor Tim McNamara at the University of Melbourne, serve as tools to assess vital communication skills among healthcare professionals trained in non-English speaking countries ( OET, 2024).

Before pursuing a healthcare career in an English-speaking country, many EFL medical undergraduates could benefit from having their communication skills assessed with a tailored assessment mechanism. Occupational English Test Speaking Assessment Criteria Glossary, 2018, which have been thoroughly tested and validated through empirical research ( Elder *et al.*, 2013; Frost *et al.*, 2015; Frost *et al.*, 2016; Pill & Knoch, 2014; Séguis & McElwee, 2019), offer a solid foundation. This widely available assessment tool assesses two main domains: Linguistic Criteria and Clinical Communication Criteria (see extended data for details; Oshimi, 2024). However, to employ the OET more extensively, assessors must receive specific training tailored to the OET’s assessment context ( O’Hagan *et al.*, 2015). Although the original OET speaking assessment criteria are well-suited for use by EFL experts, they appear overly intensive and impractical for preliminary assessments of EFL medical students in formative settings, as physicians conducting these assessments are often unfamiliar with the specific expressions and terminology the criteria require.

This research was conducted to fill this gap and surmount this challenge. Drawing inspiration from the foundational criteria of the OET speaking sub-test and assimilating insights from clinical communication experts in English-speaking countries, the objective was to develop strict and objective criteria to assess the English history-taking proficiency of EFL medical undergraduates. Particularly crucial in this pursuit is the creation of an assessment tool tailored for clinicians tasked with teaching history-taking skills in English while ensuring that it places minimal linguistic demands on assessors.

In essence, the aim of this research was to create a physician-friendly rubric inspired by the OET speaking sub-test to validly and reliably assess the English history-taking skills of EFL medical undergraduates.

## Methods

### Ethical considerations and participant consent

The research was conducted under International University of Health and Welfare (IUHW) Ethics Committee Approval Number 16-Io-246, dated April 5 ^th^, 2017. The research ethics committee endorsed access to the assessment data, and informed consent was obtained in written form from all 134 study participants.

### Origins of the assessment framework

This rubric’s conception traces its foundation to the OET speaking sub-test (see Appendix 1 in the extended data for details), a recognized and influential benchmark in assessing English proficiency within a clinical setting ( Elder *et al.*, 2013; Frost *et al.*, 2015; Frost *et al.*, 2016; Pill & Knoch, 2014; Séguis & McElwee, 2019). The specific points of the OET assessment framework are summarized below to illustrate the genesis of the newly proposed rubric.

***Linguistic criteria***

All components utilize a 0–6 scale.

- **Intelligibility**: Measures speech clarity, including pronunciation and prosodic features. A score of 6 denotes impeccable pronunciation and prosody, while lower scores reflect increasing L1 accent influence, with 0 being non-responsive.
- **Fluency**: Assesses speech flow and tempo. A score of 6 indicates seamless speech, whereas scores closer to 0 reflect disruptions such as frequent pauses or repetitions.
- **Appropriateness of language**: Assesses appropriate register, tone, and vocabulary use. Perfect appropriateness earns a 6, with decreasing scores indicating increasing inappropriateness.
- **Resources of grammar and expression**: A high score signifies mastery over a diverse range of grammar and vocabulary patterns, while lower scores point to limited vocabulary or grammatical errors.

***Clinical communication criteria***

All components utilize a 0–3 scale, with 0 defined as ineffective and 3 as adept.

- **Relationship building**: Measures skills in initiating interactions, displaying empathy, and understanding the patient's emotions, including aspects like appropriate greetings, and showing respect.
- **Understanding and incorporating the patient's perspective**: Assesses the clinician's ability to capture and address the patient's concerns, including effective explanation and cue recognition.
- **Providing structure**: Reviews the organization and flow of the interview, emphasizing sequence, topic signposting, and structuring techniques.
- **Information gathering**: Assesses skills in collecting and clarifying patient data, emphasizing active listening, open-ended questions, and summarization.
- **Information giving**: Analyzes proficiency in conveying information to patients, with emphasis on assessing patient knowledge, ensuring comprehension, and inviting feedback.

Drawing from these comprehensive parameters of the OET, the researcher aims to design a robust assessment tool tailored to the needs of EFL medical undergraduates for history-taking skills. The linguistic and clinical communication criteria, which are already established assessment points used in the OET rubric, were adapted with modifications to enhance clarity and objectivity, making them more user-friendly for clinicians involved in the assessment process.

### History-taking-centric rubric adaptation

The development of a rubric to assess the history-taking abilities of EFL medical undergraduates was undertaken by four faculty members, including the researcher, all of whom are EFL physicians specializing in teaching medical communication skills in English in Japan. With combined teaching experience ranging from two to 15 years, the team brought both linguistic and clinical expertise to the process. The researcher played a leading role in coordinating the adaptation.

The rubric was developed systematically over three months (April to June 2022), following four main stages: initial brainstorming, drafting, refining through iterative discussions, and finalizing. These discussions ensured that the rubric was comprehensive and tailored to the specific needs of history-taking assessments in EFL contexts.

To adapt the rubric from the OET criteria, careful consideration was given to the relevance of each component. The original OET “Information Giving” category was excluded, as medical undergraduates in history-taking scenarios rarely provide diagnostic information to patients. Conversely, new sections, such as Medical Interview criteria and Global Rating criteria, were introduced. These additions align with elements commonly assessed in objective structured clinical examinations (OSCE) in Japan and other EFL countries, ensuring broader applicability and relevance.

The adaptation aimed to create a rubric that is practical for clinicians while maintaining linguistic and clinical rigor. These changes reflect a thoughtful modification of the OET criteria to meet the unique demands of assessing history-taking skills in EFL settings. Major adaptations include:

- **Linguistic criteria 1–4 (L1–4)**: Streamlined descriptors for enhanced clarity.
  ○   L1: Intelligibility
    ○   L2: Fluency
    ○   L3: Appropriateness of Language
    ○   L4: Resource of Grammar and Expression
- **Clinical communication criteria 1–4 (C1–4)**: Changed from item selection to a checklist format to focus on objectivity and clarity.
  ○   C1: Relationship Building
    ○   C2: Patient’s Perspective
    ○   C3: Providing Structure
    ○   C4: Information Gathering
- **Medical interview criteria 1–3 (M1–3)**: Incorporated strategic elements such as patient safety, medical history components, and comprehensiveness of history of present illness.
  ○   M1: Patient Safety
    ○   M2: Medical History Components
    ○   M3: Comprehensiveness of History of Present Illness
- **Global rating 1–2 (G1–2)**: Introduced a comprehensive assessment comparing students’ abilities to those of their counterparts in English-speaking countries.
  ○   G1: Quality
    ○   G2: Overall Performance

Patient safety is consistently featured in OSCE checklists in Japan, underscoring its importance. The emphasis on the history of present illness is derived from its critical role in generating differential diagnoses and guiding medical decision-making. This significance is buttressed by Skeff’s findings, asserting that the history of present illness (HPI) is pivotal for an in-depth analysis of a patient’s condition ( Skeff, 2014). The Global Rating category provides a comprehensive assessment of a student’s skills in comparison to their counterparts in English-speaking countries.

The adapted rubric was named the LCM Rubric Version 1, with the acronym "LCM" denoting the three pillars of the rubric: Linguistic, Clinical Communication, and Medical Interview. Details are provided in **Appendix 2 of the extended data** ( Oshimi, 2024).

### Assessment

In July 2022, all 134 sixth-year medical undergraduates at the International University of Health and Welfare (IUHW) in Japan participated in an assessment of their English history-taking skills, which was also an integral component of their mandatory post-clinical-clerkship objective structured clinical examination (post-CC OSCE). No minors were included in this assessment.

During the first two years of their medical education, these students were extensively trained in medical subjects taught in English. This foundation was bolstered by a 120-hour medical English course tailored to enhance their English history-taking proficiency. As an ongoing measure of their English language proficiency, they had sat the Test of English as a Foreign Language Institutional Testing Program (TOEFL ITP) seven times before their post-CC OSCE assessment.

The 12-minute English history-taking assessment was structured around a simulated patient setting to replicate a realistic medical scenario. A total of eight individuals acted as simulated patients: six were IUHW faculty members, and two were volunteers, including a single native English speaker. The inter-simulated patient reliability was also statistically assessed regarding the potential bias introduced by faculty members acting as simulated patients. Although using only one native English speaker might be perceived as a limitation, given the emphasis on English history-taking skills, this composition was chosen to replicate a diverse patient base that mirrors more closely the non-Japanese patient population residing in Japan who are likely to seek medical care. Each simulated patient received individualized training, lasting one hour, to consistently play the role of a patient suffering from a headache, ultimately diagnosed as a migraine (see **Appendix 3** of the extended data for details of the patient prompts; Oshimi, 2024).

To comprehensively document each student’s performance, each simulated consultation room was fitted with two ceiling-embedded video cameras, capturing both the student and the simulated patient from dual angles. The video recordings of satisfactory visual and audio quality were the primary means for assessors to gauge the students’ performance.

The IUHW School of Medicine curated a group of 14 assessors from its broad professional connections. These assessors possess clinical education backgrounds in English-speaking countries. They were remunerated for their involvement in the assessment as external, objective third parties. The researcher did not have access to the full, personal details regarding these assessors and simulated patients and was thus unable to contact them individually after they had finished the work they had been contracted to do. As a result, authorization to access the post-CC OSCE scores of the students for the purpose of conducting this research had to be obtained from the institutional ethics committee.

Their assessments, primarily centered on the Global Rating of the LCM Rubric Version 1, were the principal metric of a student’s abilities. This Global Rating consists of two parts:

- **Global rating 1 (G1)**: Assessors checked if students engaged in clinically appropriate behaviors such as patient-centered interviewing, respect towards the patient, rapport-building, and professional conduct during the medical interview.
- **Global rating 2 (G2)**: Assessors rated students based on predefined levels of expertise, ranging from the level of an outstanding student who has finished their core clinical clerkships to that of a novice who has yet to begin their clinical clerkship.

To facilitate the assessment process, each pair of assessors reviewed the performances of 19 to 20 students using video recordings accessible on a cloud platform. The LCM Version 1 was provided via Google Forms to ensure a consistent assessment approach. This rubric, familiar to the students beforehand, highlighted the essential Medical Interview Components they were expected to cover. Furthermore, assessors were directed to consult an external resource ( Benchmark Education Solutions, 2023) for an in-depth understanding of the linguistic and clinical communication criteria embedded within the rubric.

### Data analysis

***Construct validity***

We evaluated the construct validity of our rubric through exploratory factor analysis (EFA). This analysis included 13 rubric items and 12 TOEFL ITP score variables, reflecting English proficiency at different stages of medical education. Using the Kaiser criterion and Promax rotation, we identified key factors that validate our rubric. It is important to note that due to optional participation in the TOEFL ITP exams after the third year, we occasionally used scores from earlier tests as substitutes to handle missing data effectively.

***Reliability***

We assessed reliability by measuring the internal consistency of the rubric and TOEFL scores (Cronbach’s α) and the agreement among different assessors (chi-squared tests for inter-rater reliability).

***Multiple regression analysis***

We conducted a multiple regression analysis using Global Rating 2 (G2) as the dependent variable. This analysis incorporated rubric scores and specific TOEFL ITP scores as independent variables, ensuring adherence to regression assumptions such as linearity, multicollinearity, and homoscedasticity.

***Significance threshold***

All statistical tests were anchored to a significance level set at *P* =0.05 (two-tailed). *P*-values below this threshold were interpreted as indicative of statistical significance. Statistical tests were conducted with SPSS Statistics 26 (IBM Corporation, Armonk, NY, USA).

## Results

### Scores on LCM Rubric Version 1

**Figure 1.**
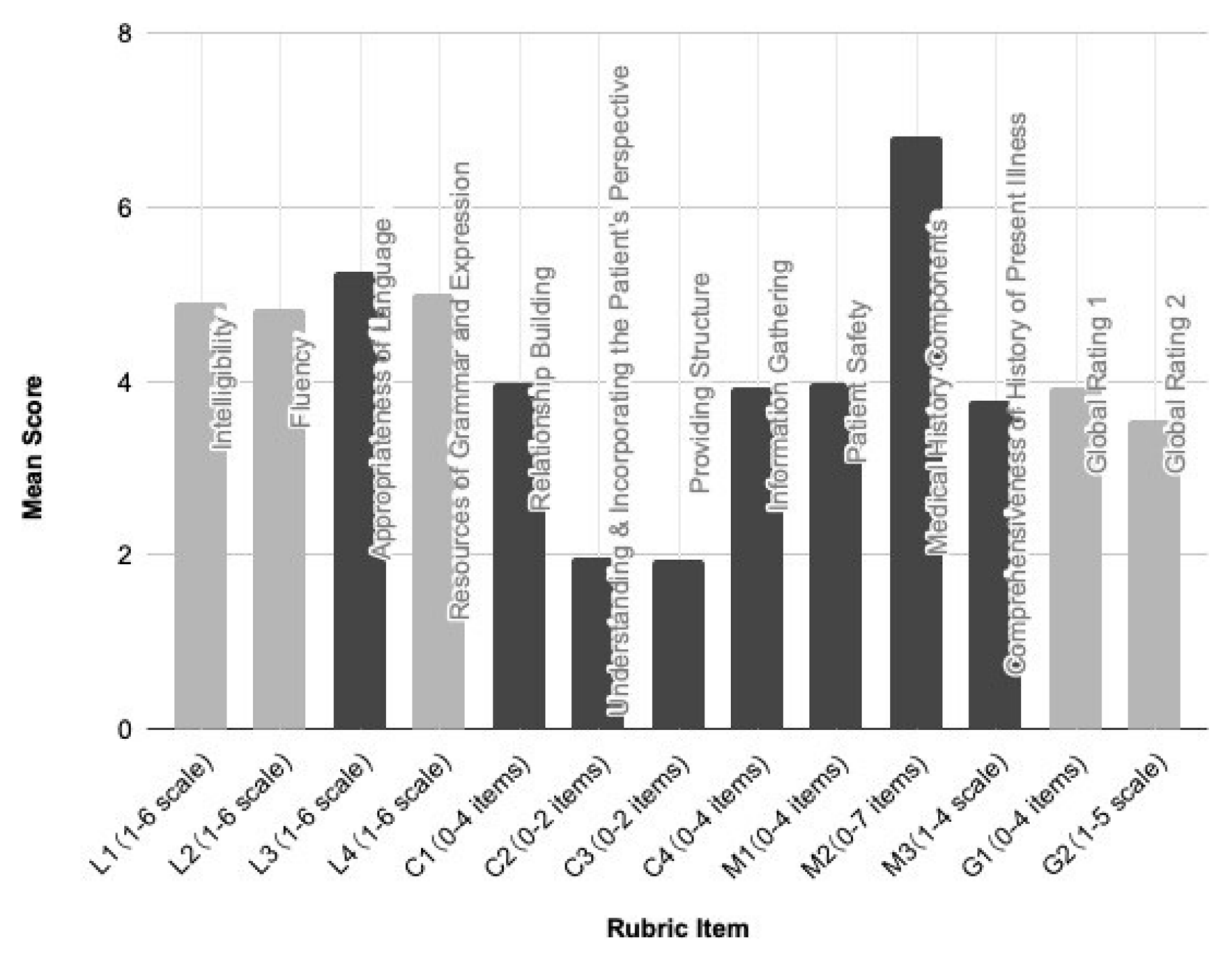
Scores on LCM Rubric Version 1. N=134 students; assessed by two assessors each.

Figure 1 presents the mean scores on the LCM Rubric Version 1.

A notable ceiling effect was discerned for eight items: L3 (Appropriateness of Language), C1 (Relationship Building), C2 (Understanding & Incorporating the Patient's Perspective), C3 (Providing Structure), C4 (Information Gathering), M1 (Patient Safety), M2 (Medical History Components), and M3 (Comprehensiveness of History of Present Illness). The prominence of this effect might stem from the rubric being revealed to examinees before the assessment, serving as an instrument guiding their preparations. Despite the absence of a floor effect, its educational implications were positive: the rubric, serving as a preparatory guideline, likely influenced the high-scoring trend.

***Construct validity***

The construct validity of the LCM Version 1 rubric was established through exploratory factor analysis (EFA). The initial analysis of 25 variables, comprising 13 rubric items and 12 TOEFL ITP scores, encountered computational challenges, particularly related to the C1 (Relationship Building) variable due to an influential outlier. Additionally, the interdependence of comprehensive and skill-specific TOEFL scales, influenced by the TOEFL scoring structure, presented challenges.

In total, three significant factors emerged when EFA was refined to 15 variables, yielding a Cronbach’s alpha coefficient of 0.690, hinting at potential variances introduced by the TOEFL ITP scores. Table 1 shows the factor loadings for each of the 12 variables against the three identified factors.

**Table 1.** Refined EFA variables and identified factors. *N*=134 students; assessed by two assessors each.

Rubric item	Identified factors		
	Factor 1	Factor 2	Factor 3
	Linguistic-clinical distinction	Communication dynamics	Medical comprehension
**L1 (1-6 scale)**	**0.882**	-0.116	0.06
**L2 (1-6 scale)**	**0.917**	-0.054	0.05
**L3 (1-6 scale)**	**0.689**	0.018	-0.157
**L4 (1-6 scale)**	**0.839**	-0.08	-0.048
**C2 (0-2 items)**	0.186	-0.019	-0.227
**C3 (0-2 items)**	-0.093	0.349	0.353
**C4 (0-4 items)**	-0.03	**0.72**	0.053
**M1 (0-4 items)**	-0.147	0.299	0.07
**M2 (0-7 items)**	-0.005	-0.083	**0.427**
**M3 (1-4 scale)**	0.107	0.066	**0.605**
**G1 (0-4 items)**	0.088	**0.742**	-0.24
**G2 (1-5 scale)**	**0.503**	0.342	0.1
**L1**: Intelligibility **L2**: Fluency **L3**: Appropriateness of Language **L4**: Resource of Grammar and Expression		**C1**: Relationship Building **C2**: Patient's Perspective **C3**: Providing Structure **C4**: Information Gathering	
**M1**: Patient Safety **M2**: Medical History Components **M3**: Comprehensiveness of HPI		**G1**: Quality **G2**: Overall Performance	
Extraction Method: Maximum Likelihood Rotation Method: Promax with Kaiser Normalization Note: Rotation converged in 4 iterations Rubric Item C1 (Relationship Building) was excluded from the analysis due to a lack of variance in the scores, with nearly all values being identical (standard deviation < 0.001). This issue caused computational challenges, as noted in the text.			

The three factors identified in the EFA are described as follows: Factor 1, labeled “ **Linguistic-Clinical Distinction**,” differentiates between linguistic aspects (L) and clinical components (C & M). Factor 2, labeled “ **Communication Dynamics**,” encompasses elements that foster doctor-patient rapport-building (G1), facilitate information gathering (C4), and include other relevant linguistic and medical tasks (L & M). Thirdly, Factor 3, labeled “ **Medical Comprehension**,” highlights the subtleties in medical history components (M2), the comprehensiveness of the history of the present illness (M3), and the interpretation of patient perspectives (C2).

The divergence between rubric items and TOEFL scores suggested an iterative EFA approach. Without TOEFL scores, the Cronbach’s alpha improved to 0.799. However, caution is advised due to skews in items like Relationship Building (C1) and potential ceiling effects in other variables.

In summary, while the EFA supports the construct validity of the LCM Rubric Version 1, nuances related to specific variables and consistency are notable. Nevertheless, the LCM Rubric Version 1 stands as a promising assessment tool.

### Reliability

***Internal consistency***

Excluding the TOEFL ITP scores and the C1 (Relationship Building) variable, which displayed no variance (standard deviation < 0.001) due to all but one score being identical, the rubric items achieved a Cronbach’s α coefficient of 0.799, indicating satisfactory reliability. Table 2 provides Cronbach’s α values for individual variables.

**Table 2.** Cronbach’s α coefficient summary. *N*=134 students; assessed by two assessors each.

Rubric item	Scale mean value	Scale variance	Item-total correlation	Squared multiple correlation	Cronbach's α
**L1 (1-6 scale)**	45.08	11.322	0.744	0.668	**0.746**
**L2 (1-6 scale)**	45.14	10.866	0.805	0.736	**0.736**
**L3 (1-6 scale)**	44.72	12.179	0.571	0.432	**0.772**
**L4 (1-6 scale)**	44.97	12.456	0.677	0.592	**0.758**
**C2 (0-2 items)**	48.01	16.207	0.084	0.062	**0.805**
**C3 (0-2 items)**	48.04	15.877	0.222	0.194	**0.801**
**C4 (0-4 items)**	46.05	15.47	0.327	0.372	**0.795**
**M1 (0-4 items)**	46.01	16.281	0.028	0.091	**0.806**
**M2 (0-7 items)**	43.19	15.616	0.127	0.139	**0.808**
**M3 (1-4 scale)**	46.21	14.753	0.377	0.254	**0.79**
**G1 (0-4 items)**	46.05	15.622	0.329	0.366	**0.796**
**G2 (1-5 scale)**	46.39	11.043	0.681	0.519	**0.757**
**L1**: Intelligibility **L2**: Fluency **L3**: Appropriateness of Language **L4**: Resource of Grammar and Expression			**C1**: Relationship Building **C2**: Patient's Perspective **C3**: Providing Structure **C4**: Information Gathering		
**M1**: Patient Safety **M2**: Medical History Components **M3**: Comprehensiveness of HPI			**G1**: Quality **G2**: Overall Performance		

While the α coefficient’s stability persisted with each variable’s exclusion, emphasizing the rubric’s integrity, its inclusion of TOEFL ITP scores reduced the coefficient to 0.690. Variables like C1 (Relationship Building) showed significant skewness, and others, including L3 (Appropriateness of Language), all of the Clinical Communication variables, and all of the Medical History variables, presented ceiling effects, warranting careful interpretation in subsequent analyses.

***Inter-rater reliability***

Rating consistency across assessors was examined for the newly added criteria: M1, M2, M3, G1, and G2. With the chi-squared test due to the categorical score data, significant variations were observed in all criteria. Specifically, two raters assessing the same 19 students showed marked differences only in the M1 criterion ( *P* =0.046). This emphasizes the variability in scoring and the importance of thorough training for consistent evaluations. Additionally, inter-simulated patient reliability mirrored the procedure for inter-rater reliability, revealing no significant differences among simulated patients.

***Multiple regression analysis***

In the regression analysis, the C1 (Relationship Building) data showed significant skewness, predominantly scoring 4 with one score of 2. Given its pronounced skewness, C1 (Relationship Building) was omitted, and no transformation was applied as it would be ineffective. The regression model had a strong correlation with a multiple correlation coefficient (R) of 0.686, and a coefficient of determination (R ^2^) of 0.471, being statistically significant ( *P* < 0.001). The F-statistic was 16.633 with degrees of freedom (df) of 13 for regression, and 243 for residuals, and the model's standard error was 0.728 ( Table 3).

**Table 3.** Multiple regression analysis results for the relationship between specific criteria and Global Rating 2 (G2) scale. *N* =134 students; assessed by two assessors each.

Predictor	B (Unstandardized coefficient)	Standard error	Beta (Standardized coefficient)	t-value	p-value	95% Confidence interval (B)	Tolerance	VIF
**Intercept** **(Constant)**	-7.178	1.796	-	-3.996	0	(-10.716, -3.640)	-	-
**L1 (1-6** **scale)**	0.103	0.093	0.09	1.113	0.267	(-0.079, 0.285)	0.33	3.031
**L2 (1-6** **scale)**	0.126	0.101	0.113	1.246	0.214	(-0.073, 0.324)	0.265	3.773
**L3 (1-6** **scale)**	0.241	0.068	0.215	3.561	**0**	(0.108, 0.374)	0.598	1.671
**L4 (1-6** **scale)**	0.287	0.099	0.211	2.914	**0.004**	(0.093, 0.482)	0.415	2.409
**C2 (0-2** **items)**	0.147	0.322	0.023	0.458	0.647	(-0.486, 0.781)	0.885	1.13
**C3 (0-2** **items)**	0.372	0.21	0.091	1.77	0.078	(-0.042, 0.785)	0.822	1.217
**C4 (0-4** **items)**	0.581	0.17	0.175	3.413	**0.001**	(0.245, 0.916)	0.826	1.21
**M1 (0-4** **items)**	0.676	0.35	0.094	1.932	0.054	(-0.013, 1.365)	0.918	1.089
**M2 (0-7** **items)**	-0.033	0.105	-0.016	-0.318	0.751	(-0.239, 0.173)	0.844	1.185
**M3 (1-4** **scale)**	0.306	0.111	0.147	2.742	**0.007**	(0.086, 0.525)	0.761	1.314
**Admission** **TOEFL ITP**	-0.002	0.002	-0.078	-0.709	0.479	(-0.006, 0.003)	0.179	5.584
**Post-English** **Ed. TOEFL** **ITP**	0.002	0.002	0.103	0.916	0.361	(-0.002, 0.006)	0.174	5.764
**Pre-History-** **taking** **TOEFL ITP**	0	0.002	-0.026	-0.241	0.81	(-0.004, 0.003)	0.184	5.446
**L1**: Intelligibility **L2**: Fluency **L3**: Appropriateness of Language **L4**: Resource of Grammar and Expression				**C1**: Relationship Building **C2**: Patient's Perspective **C3**: Providing Structure **C4**: Information Gathering				
**M1**: Patient Safety **M2**: Medical History Components **M3**: Comprehensiveness of HPI				**G1**: Quality **G2**: Overall Performance				

In total, four specific criteria emerged with a statistically significant relationship with the dependent variable G2 (Global Rating 2), which assesses students’ clinical competency progression through core clinical clerkships such as L3 (Appropriateness of Language), L4 (Resources of Grammar and Expression), C4 (Information Gathering), and M3 (Comprehensiveness of History of Present Illness).

Despite their incorporation into the model, TOEFL ITP scores did not show a statistically significant relationship with the dependent variable. The finding that TOEFL ITP scores did not fall below the significance threshold underscores the idea that the rubric assesses skills distinct from those gauged by a standard English language proficiency test like the TOEFL ITP.

## Discussion

The aim of this study was to create a specialized rubric to assess the English history-taking skills of EFL medical undergraduates. Drawing inspiration from the OET speaking sub-test, the rubric was developed and validated through a comprehensive empirical study.

The analysis of mean scores across the LCM Rubric Version 1 criteria revealed a pronounced ceiling effect in eight items. This finding suggests that students may have been well-prepared, potentially due to prior exposure to the rubric, which could have guided their preparation effectively. This outcome highlights the dual role of the rubric as both an assessment tool and a teaching resource. Furthermore, the high scores may also reflect the robust educational standards achieved by most students. However, the concentration of high scores necessitates further examination of potential external factors, such as assessor leniency or the influence of structured preparation, which may have contributed to this effect.

Construct validity, assessed through exploratory factor analysis, revealed three distinct factors: linguistic-clinical distinction, communication dynamics, and medical comprehension. these factors underscore the importance of differentiating linguistic proficiency from clinical communication skills, highlighting the LCM Rubric Version 1’s specialized nature. It is worth noting that the inclusion of the TOEFL ITP scores affected internal consistency, as reflected by a sub-threshold Cronbach’s alpha value of 0.690. validating the rubric’s specific focus.

While the rubric demonstrated strong internal consistency (Cronbach’s α coefficient of 0.799), the discrepancies in assessor scores, particularly for the C1 (Relationship Building) variable, highlight the need to standardize assessments. The development of simplified educational materials, such as sample videos illustrating various performance levels and corresponding rubric scores, would be necessary. These materials aim to improve assessors' understanding of the rubric and ensure consistent assessments.

The multiple regression analysis underscored four vital criteria linked with the G2 (Global Rating 2) assessment of clinical competency. Among these, M3 (Comprehensiveness of History of Present Illness) stood out with marked significance, emphasizing the necessity to prioritize training in comprehensive history of present illness (HPI) for EFL medical students, enhancing their clinical competencies and ultimately patient care outcomes. While it is common for novice medical students to lean on standardized expressions, the depth and intricacy of HPI must be considered. Hence, EFL undergraduate curricula in medical English communication should prioritize an exhaustive exploration of HPI. Moreover, while other criteria like L3 (Appropriateness of Language) and L4 (Resources of Grammar and Expression) also have a bearing on performance, it is noteworthy that the LCM Rubric’s assessment method is markedly different from that of general English proficiency tests, as evidenced by the non-significant relationship with TOEFL ITP scores.

In summary, the LCM Rubric Version 1, informed by the OET speaking sub-test criteria, provides a tailored approach for assessing the English history-taking skills of EFL medical undergraduates. The findings about the rubric's validation and reliability suggest its potential utility in educational settings, although the results should be interpreted with caution due to the study's limitations. They bear implications for curriculum development and student training in global medical communication.

### Limitations

This study has a number of limitations. Firstly, it examines the English history-taking abilities of EFL medical undergraduates at a single private Japanese medical school. The unique emphasis on English at IUHW may limit the findings' generalizability to other academic settings. During the first two years of their medical education, IUHW students were extensively trained in medical subjects taught in English. This foundation was bolstered by a 120-hour medical English course specifically designed to enhance their English history-taking proficiency. Given its primary focus on Japan, it is also crucial to consider linguistic nuances in different international medical contexts.

Relying only on the OET speaking sub-test might omit some aspects of students' linguistic proficiency. The selection of assessors, despite their diversity, introduces potential bias, and limited data on them could affect the study's depth. The specific focus on migraines and a particular cohort may not capture the broader EFL medical experience. Video recordings, while practical, might only partially capture face-to-face interactions. Concerns about sample representativeness and the influence of access to post-CC OSCE scores on the study’s objectivity are worth noting.

### Recommendations for further research

Further research involving other institutes in Japan and overseas is necessary to investigate the rubric's applicability to contexts beyond that of the single institution featured in the current study. Integrating artificial intelligence with the current rubric could optimize assessments in terms of efficiency and cost. Feedback from assessors on the LCM Rubric Version 1 will further inform assessment refinement. Although this research provides valuable insights into the English history-taking abilities of EFL medical undergraduates at IUHW, the highlighted limitations set the direction for future studies.

## Data Availability

Given the sensitive nature of the data, especially considering that it pertains to student grades and the involvement of external assessors, the institutional ethics committee of the International University of Health and Welfare (IUHW) mandated stringent controls over data access. In alignment with our commitment to ensuring the confidentiality and protection of participant data, particularly the post-CC OSCE grades, the raw data from this study is not publicly accessible. Participants provided written consent to use their grades strictly for research purposes, not including public dissemination. This approach fully complies with the data protection concerns highlighted by the Institutional Review Board (IRB) at IUHW, which emphasizes stringent measures for data privacy and ethical handling of sensitive information. To maintain the integrity of the research and honor our confidentiality commitments, we have established a structured process for accessing the data under specific, ethically approved conditions, as also reviewed and sanctioned by the IUHW Research Ethics Committee. The process is as follows: Researchers interested in accessing the data should initially contact the corresponding author at oshimi@iuhw.ac.jp with a comprehensive explanation of the data's intended use. Subsequently, an official request must be submitted to the IUHW Research Ethics Committee. This request should articulate a transparent and ethical purpose for data usage, aligning with the study's objectives and the committee's ethical standards. Conditional access to the data may be granted based on the committee's review, ensuring that the request strictly adheres to the ethical guidelines and the study's objectives. This structured approach underscores our adherence to the guidelines stipulated by the IRB. It demonstrates our unwavering commitment to upholding data privacy while facilitating ethically sound research that aligns with the study's purpose. Appendix 1 (SPEAKING Assessment Criteria and Level Descriptors) is publicly available by the OET. For access to the full document, please visit the provided URL: https://prod-wp-content.occupationalenglishtest.org/resources/uploads/2018/08/22102547/speaking-assessment-criteria-updated-2018.pdf. Zenodo: Developing a clinician-friendly rubric for assessing history-taking skills in medical undergraduates speaking English as a foreign language. https://zenodo.org/records/10610376 ( Oshimi, 2024). This project contains the following extended data: Appendix 2.pdf (LCM Rubric Version 1) Appendix 3.pdf (Patient Prompts) Data are available under the terms of the Creative Commons Attribution 4.0 International license (CC-BY 4.0).
