# Supplementary material for: Developing a clinician-friendly rubric for assessing history-taking skills in medical undergraduates speaking English as a foreign language
Source: MedEdPublish (2016). 2025 Aug 28;14:11. Originally published 2024 Mar 8. [Version 3] doi: 10.12688/mep.19911.3 (PMC11555326; doi:10.12688/mep.19911.3)
Supplement: Peer review history

# Reviewer report: Reviewer response for version 3

**DOI:** 10.21956/mep.22339.r43302

**Published:** 2025-09-03

**Authors:** Alexandra Lopez Vera

**Affiliations:** 1 California University of Science and Medicine, Colton, California, USA

The authors have addressed my previous reservations. Typos are corrected; an appropriate OET citation and context are added; the rubric development process is described with clear rationale for excluding “Information Giving” and adding “Medical Interview” and “Global Rating”; all criteria are now named and defined; the variable set is clarified (13 rubric items + 12 TOEFL ITP scores) and the factor count corrected to three; the omission of C1 is explained (near-zero variance); and the emphasis on developing educational materials (e.g., sample videos) aligns with the study’s goals. The validation and reliability sections are clearer and consistent with the stated aims. I have no further concerns.

Have any limitations of the research been acknowledged?

Partly

Is the work clearly and accurately presented and does it cite the current literature?

Yes

If applicable, is the statistical analysis and its interpretation appropriate?

Partly

Are all the source data underlying the results available to ensure full reproducibility?

Yes

Is the study design appropriate and is the work technically sound?

Yes

Are the conclusions drawn adequately supported by the results?

Yes

Are sufficient details of methods and analysis provided to allow replication by others?

Yes

Reviewer Expertise:

Medical Spanish and Public Health

I confirm that I have read this submission and believe that I have an appropriate level of expertise to confirm that it is of an acceptable scientific standard.

# Reviewer report: Reviewer response for version 3

**DOI:** 10.21956/mep.22339.r43303

**Published:** 2025-09-02

**Authors:** Dan Douglas

**Affiliations:** 1 Iowa State University, Ames, Iowa, USA

The revisions to the earlier draft are well done and the article is now ready for indexing.

Have any limitations of the research been acknowledged?

Yes

Is the work clearly and accurately presented and does it cite the current literature?

Yes

If applicable, is the statistical analysis and its interpretation appropriate?

Partly

Are all the source data underlying the results available to ensure full reproducibility?

Yes

Is the study design appropriate and is the work technically sound?

Yes

Are the conclusions drawn adequately supported by the results?

Yes

Are sufficient details of methods and analysis provided to allow replication by others?

Yes

Reviewer Expertise:

Language assessment, languages for specific purposes.

I confirm that I have read this submission and believe that I have an appropriate level of expertise to confirm that it is of an acceptable scientific standard.

# Reviewer report: Reviewer response for version 2

**DOI:** 10.21956/mep.22075.r40006

**Published:** 2024-11-19

**Authors:** Dan Douglas

**Affiliations:** 1 Iowa State University, Ames, Iowa, USA

**General Comments:**

 This report on a study to develop a more specific purpose rubric for assessing the medical communication skills of undergraduate, i.e. novice medical students, is based on a sound methodology and a carefully conducted procedure.  The conclusions drawn from the data are suitably cautious and appropriate, and the limitations of the study are honestly described.  Finally, the suggestions for further research are appropriate.  I have a few relatively minor comments, queries, and suggestions for the author to consider.  It may be that some of my questions about the data are due to my own misreading, so take them with a grain of salt!

**Specific Comments:**

 p. 3, para 1: Typo: accuracy

 p. 3, para 3: Provide a citation for the OET: www.occupationalenglishtest.org

 p. 3, para 4: “…its application seems too intensive for preliminary evaluations of EFL medical students in contexts such as formative assessments to refine their English history-taking skills.” Is there any evidence for this assertion?  Or perhaps you could provide an additional sentence or two explaining in what way the OET criteria are too intensive.

 p. 4, right column, para 3: LCM - You explain the acronym below, but readers will want to know what LCM denotes here, I think.

 p. 4, right column, para 3: Major Adaptations - So, there were 4 linguistic criteria, 4 clinical communication criteria, 3 medical interview criteria and 2 global ratings.  You name them in Fig. 1 below, but perhaps you could list them here as readers will want to know.

 p. 6, left column, para 4: 25 variables - I'm sorry, I'm finding it difficult to follow your discussion: what are the 25 variables?  They don’t seem to be mentioned prior to this paragraph.

 p. 6, right column, para 2: “…four significant factors emerged…” You mention four factors here, then “three identified factors” at the end of the paragraph…

 p. 7, Table 1: Rubric Item C1 is omitted from the table.  Earlier, you mention that C1 showed an “influential outlier” but don’t explain why it was omitted from the table until later in the paper.  I think readers will wonder…could you put in a note explaining the omission?

 p. 7, left column, para 1: “…skews in items like Relationship Building (C1)…” Is there data showing this skewness?  Simply saying it was skewed and omitting C1 from the analysis isn’t enough, I think.

 p. 8, right column, para 2: Typo: *The* aim of this study…

 p. 8, right column, para 3: “…the concentration of high scores prompts reflection on assessor leniency or the influence of preparation.”  Assuming the rubric was "good" in the sense that it taps into the best aspects of history taking, preparation based on it would be beneficial; however, the opposite may also be true...

 p. 9, right column, para 1: Typo: *t*he discrepancies…

 p. 9, right column, para 1: “…underscore the need for rigorous assessor training to ensure consistent assessments.”  True. Yet one of the reasons for carrying out the study was to reduce the need for "rigorous assessor training."  On p. 3 you say "...While such specialized training is apt for decisions concerning hiring healthcare professionals, its application seems too intensive for preliminary evaluations of EFL medical students..." Is the current recommendation contradictory? Perhaps some clarification is needed.

Have any limitations of the research been acknowledged?

Yes

Is the work clearly and accurately presented and does it cite the current literature?

Yes

If applicable, is the statistical analysis and its interpretation appropriate?

Partly

Are all the source data underlying the results available to ensure full reproducibility?

Yes

Is the study design appropriate and is the work technically sound?

Yes

Are the conclusions drawn adequately supported by the results?

Yes

Are sufficient details of methods and analysis provided to allow replication by others?

Yes

Reviewer Expertise:

Language assessment, languages for specific purposes.

I confirm that I have read this submission and believe that I have an appropriate level of expertise to confirm that it is of an acceptable scientific standard, however I have significant reservations, as outlined above.

# Response

**Published:** 2024-12-27

**Authors:** TAKAYUKI OSHIMI

**Affiliations:** School of Medicine, International University of Health and Welfare, Narita, Chiba, Japan

Thank you for your thoughtful review and positive feedback. I appreciate your insightful suggestions and have addressed your specific comments below.

**Reviewer comments: Specific Comments** **p. 3, para 1: Typo: accuracy**

**Author response: **The typo has been corrected. Thank you for catching this error.

**Reviewer comments: p. 3, para 3: Provide a citation for the OET: www.occupationalenglishtest.org**

**Author response: **Thank you for your suggestion. The citation has been added, and the text has been revised as follows:

 "Occupational English Test (OET), developed by Professor Tim McNamara at the University of Melbourne (OET ©️2024), serves as a tool to assess vital communication skills among healthcare professionals trained in non-English speaking countries (Mladenovic et al. 2022)." The reference has also been included in the references section.

**Reviewer comments: p. 3, para 4: “…its application seems too intensive for preliminary evaluations of EFL medical students in contexts such as formative assessments to refine their English history-taking skills.”**

**Author response: **Thank you for highlighting this. Additional clarification has been provided to explain the intensity of the original OET criteria and their limited practicality in preliminary settings. The revised text clarifies that the criteria are designed for EFL experts, making them overly complex for clinicians unfamiliar with the specific terminology and expressions.

**Reviewer comments: p. 4, right column, para 3: LCM - You explain the acronym below, but readers will want to know what LCM denotes here, I think.**

**Author response: **Thank you for your feedback. The acronym "LCM" has been removed from this section for clarity, and the updated text now reads:

 "The adaptation process aimed to make the criteria more understandable and applicable to clinicians. The following points showcase the changes from the original structure of the OET. Major adaptations include:"

**Reviewer comments: p. 4, right column, para 3: Major Adaptations - So, there were 4 linguistic criteria, 4 clinical communication criteria, 3 medical interview criteria and 2 global ratings.**

**Author response: **Thank you for your suggestion. The text has been revised to include the names and descriptions of all criteria for better clarity.

**Reviewer comments: p. 6, left column, para 4: 25 variables - I'm sorry, I'm finding it difficult to follow your discussion: what are the 25 variables?**

**Author response: **Thank you for pointing this out. The text has been revised to clearly describe the 25 variables, which include 13 rubric items and 12 TOEFL ITP scores, and their role in validating the rubric’s construct.

**Reviewer comments: p. 6, right column, para 2: “…four significant factors emerged…”**

**Author response: **Thank you for catching this error. It was indeed three factors, not four. The text has been corrected accordingly.

**Reviewer comments: p. 7, Table 1: Rubric Item C1 is omitted from the table. Earlier, you mention that C1 showed an “influential outlier” but don’t explain why it was omitted from the table until later in the paper.**

**Author response: **Thank you for your comment. A note has been added to Table 1 explaining that C1 was excluded due to its lack of variance (standard deviation < 0.001), which caused computational challenges during the analysis. Additionally, the text now includes an explanation of this issue and its impact.

**Reviewer comments: p. 7, left column, para 1: “…skews in items like Relationship Building (C1)…” Is there data showing this skewness?**

**Author response: **Thank you for your observation. The text has been revised to clarify the skewness issue with C1 and provide data supporting its exclusion.

**Reviewer comments: p. 8, right column, para 2: Typo: The aim of this study…**

**Author response: **Thank you for pointing this out. The entire sentence has been revised for clarity and accuracy.

**Reviewer comments: p. 8, right column, para 3: “…the concentration of high scores prompts reflection on assessor leniency or the influence of preparation.”**

**Author response: **Thank you for raising this important point. The revised text now discusses both the positive role of the rubric as a teaching and assessment tool and the potential influence of external factors, such as preparation and assessor leniency, on the concentration of high scores.

**Reviewer comments: p. 9, right column, para 1: Typo: the discrepancies…**

**Author response: **The typo has been corrected. Thank you for identifying this.

**Reviewer comments: p. 9, right column, para 1: “…underscore the need for rigorous assessor training to ensure consistent assessments.”**

**Author response: **Thank you for your comment. To address the potential contradiction, the text has been revised to emphasize the development of educational materials, such as sample videos, rather than rigorous training. This approach aligns with the study’s aim to minimize linguistic demands on assessors while improving consistency.

# Reviewer report: Reviewer response for version 2

**DOI:** 10.21956/mep.22075.r39697

**Published:** 2024-11-11

**Authors:** Laely Hidayati

**Affiliations:** 1 Universitas Brawijaya, Malang, Indonesia

The article describes the creation and validation of a clinician-friendly rubric for assessing the English history-taking skills of EFL (English as a Foreign Language) medical undergraduates. Inspired by the OET speaking sub-test, the rubric aims to provide a reliable assessment tool that requires minimal linguistic training for assessors. The study conducted exploratory factor analysis and multiple regression analyses to validate the rubric's construct and reliability, yielding three main assessment factors: linguistic-clinical distinction, communication dynamics, and medical comprehension. Limitations are acknowledged, particularly regarding sample diversity and institutional context.

**Suggestion:**

 1. Although the testing phase is well-detailed, more clarity is needed on the initial rubric development process. Specifically, the adaptation process involving four faculty members could be expanded for replicability.

 2. Expanding on how specific criteria were selected and refined would help other institutions adopt this rubric.

 3. If possible, providing anonymized data or expanded summary statistics could improve reproducibility while maintaining ethical standards.

Have any limitations of the research been acknowledged?

Yes

Is the work clearly and accurately presented and does it cite the current literature?

Yes

If applicable, is the statistical analysis and its interpretation appropriate?

Yes

Are all the source data underlying the results available to ensure full reproducibility?

Partly

Is the study design appropriate and is the work technically sound?

Yes

Are the conclusions drawn adequately supported by the results?

Yes

Are sufficient details of methods and analysis provided to allow replication by others?

Partly

Reviewer Expertise:

English for Specific Purposes, English for Medical Purpose, and English Language Teaching

I confirm that I have read this submission and believe that I have an appropriate level of expertise to confirm that it is of an acceptable scientific standard, however I have significant reservations, as outlined above.

# Response

**Published:** 2024-12-27

**Authors:** TAKAYUKI OSHIMI

**Affiliations:** School of Medicine, International University of Health and Welfare, Narita, Chiba, Japan

**Reviewer Comments: 1. Although the testing phase is well-detailed, more clarity is needed on the initial rubric development process. Specifically, the adaptation process involving four faculty members could be expanded for replicability.**

**Author Response: **Thank you for your valuable comment. To address your feedback, I have expanded the description of the rubric development process, detailing the main stages (brainstorming, drafting, refining, and finalizing) conducted over three months. I also clarified the adaptation process, including excluding the "Information Giving" category and introducing new sections like Medical Interview criteria and Global Rating criteria, to align the rubric with history-taking scenarios in EFL contexts. 

**Reviewer Comments: 2. Expanding on how specific criteria were selected and refined would help other institutions adopt this rubric.**

**Author Response: **Thank you for your insightful comment. I have expanded the description of how specific criteria were selected and refined, including the rationale for excluding the "Information Giving" category and introducing new sections, such as Medical Interview and Global Rating criteria. These changes were based on their relevance to history-taking scenarios and alignment with commonly assessed elements in EFL countries, ensuring the rubric's broader applicability. 

**Reviewer Comments: 3. If possible, providing anonymized data or expanded summary statistics could improve reproducibility while maintaining ethical standards.**

**Author Response: **Thank you for your thoughtful comment. The " **Data availability**" section outlines explicitly the measures taken to ensure confidentiality and ethical handling of sensitive data, including student grades and external assessor involvement. As the institutional ethics committee mandated, the raw data cannot be publicly accessible. However, I have established a structured process for requesting data access under ethically approved conditions. This approach, detailed in the " **Data availability**" section, balances the need for data privacy with the potential for reproducibility. I appreciate your understanding of these necessary measures.

# Reviewer report: Reviewer response for version 2

**DOI:** 10.21956/mep.22075.r39666

**Published:** 2024-11-06

**Authors:** Thomas Mayers

**Affiliations:** 1 Institute of Medicine, University of Tsukuba, Tsukuba, Japan

I am happy that the author has answered my concerns satisfactorily.

 I am happy to approve this version of the manuscript.

Have any limitations of the research been acknowledged?

Partly

Is the work clearly and accurately presented and does it cite the current literature?

Yes

If applicable, is the statistical analysis and its interpretation appropriate?

Yes

Are all the source data underlying the results available to ensure full reproducibility?

Partly

Is the study design appropriate and is the work technically sound?

Yes

Are the conclusions drawn adequately supported by the results?

Yes

Are sufficient details of methods and analysis provided to allow replication by others?

Partly

Reviewer Expertise:

NA

I confirm that I have read this submission and believe that I have an appropriate level of expertise to confirm that it is of an acceptable scientific standard.

# Reviewer report: Reviewer response for version 1

**DOI:** 10.21956/mep.21327.r38048

**Published:** 2024-08-16

**Authors:** Thomas Mayers

**Affiliations:** 1 Institute of Medicine, University of Tsukuba, Tsukuba, Japan

**Developing a clinician-friendly rubric for assessing history-taking skills in medical undergraduates speaking English as a foreign language**

 In this study, Oshimi describes the development and validation of a “physician-friendly rubric” for use when assessing the medical history-taking skills of medical students in contexts where English is a foreign language. The rubric is designed around the Occupational English Test speaking sub-test. The rubric was tested by 14 physicians who used it to assess 134 Japanese students. The study successfully describes the procedures for the testing of the rubric. Equipping medical students with the skills to care for English-speaking patients is very important and having reliable ways to assess that ability is likewise vital. I commend the author for his work in this field.

 While I think the manuscript is largely complete, I have a number of concerns that I would like to raise and suggestions to make for its improvement.

- Firstly, I feel that while the testing of the rubric is well described, its initial creation process seems to be somewhat vague. In the section “Origins of the assessment framework” it is not clear whether the linguistic criteria and clinical communication criteria are extracted from the OET by the author or already established assessment points (rubric) already used for the OET.
- Next the process of “adapting a rubric” seems to state that it was done by 4 people. I wonder why they are not listed as authors on the paper if they have created and tested the rubric.
- No timeframe is given as to when the rubric was made and how long it took to make.
- I think the major limitation of this paper is the fact that the rubric was tested at a single institution in Japan; therefore, it is impossible to state, “In summary, the LCM Rubric Version 1, informed by the OET speaking sub-test criteria, provides a tailored approach for assessing the English history-taking skills of EFL medical undergraduates.” I think you have to temper the statement here a little, and in the limitations section, state clearly that the data are derived from a single institution in Japan, which limits the generalizability of the findings. 

**Minor concerns:**

- What does the author mean by “equitable” in reference to the assessment criteria for EFL medical students? Maybe “stringent and objective criteria” would be a more appropriate?
- The information in following sentence seems to be a repetition of that given in the preceding sentences. “This adaptive approach was made necessary by the elective status of the TOEFL ITP following the third year of their study.”
- **In the Results section the authors often refer to items in the rubric by an alpha-numeric identifier such as in this sentence:** “A notable ceiling effect was discerned for eight items: L3, C1, C2, C3, C4, M1, M2, and M3.” This is not very helpful for the reader, as they have to constantly refer to the tables to understand what these identifiers refer to. I think it would be better to write them out in full throughout the text.
- **This sentence sounds unnatural:** “The adapted rubric focused on history-taking is termed the LCM Rubric Version 1.” How about something like “The adapted rubric was named the LCM Rubric Version 1, with the acronym "LCM" denoting the three pillars of the rubric: Linguistic, Clinical Communication, and Medical Interview.” 
- **The English grammar here is confusing: **“The study examining English history-taking abilities of EFL medical undergraduates at a private Japanese medical school has limitations.” Something like this would be better: “This study has a number of limitations. Firstly…”.
- **You state the following: **“The unique emphasis on English at IUHW may limit the findings' generalizability to other academic settings.” You might need to clarify the “unique emphasis on English at IUHW” for the reader by comparing it to the English language training at a typical Japanese medical school curriculum.
- **I think the following sentence is AI generated: **“This study underscores the importance of expansive inter-institutional collaboration between medical schools in Japan and their international counterparts to achieve a richer, more diverse participant base.” In light of my comment above, I think it would be better to say something like, for example: “To further investigate the applicability of the rubric to contexts beyond that of the single institution featured in the current study, further research involving other institutes in Japan and overseas is necessary.”
  This is a stylistic point, but in the examples below, you give agency to things that are not alive: “This study aimed to create…” “This research endeavors to…”  “this research aims to devise…” “the present research aims to design…”
  The researcher has the human agency to create, devise, endeavor, design, while a study or research cannot. So, it is better to express this something like: “The aim of the study was…” “The purpose of this research was…”
- Finally, I suspect that ChatGPT has been used in the creation of this manuscript. If so, it should be acknowledged in the manuscript. I also suggest a native English-speaker check.

Have any limitations of the research been acknowledged?

Partly

Is the work clearly and accurately presented and does it cite the current literature?

Yes

If applicable, is the statistical analysis and its interpretation appropriate?

Yes

Are all the source data underlying the results available to ensure full reproducibility?

Partly

Is the study design appropriate and is the work technically sound?

Yes

Are the conclusions drawn adequately supported by the results?

Yes

Are sufficient details of methods and analysis provided to allow replication by others?

Partly

Reviewer Expertise:

Medical Education

I confirm that I have read this submission and believe that I have an appropriate level of expertise to confirm that it is of an acceptable scientific standard, however I have significant reservations, as outlined above.

# Response

**Published:** 2024-08-24

**Authors:** TAKAYUKI OSHIMI

**Affiliations:** School of Medicine, International University of Health and Welfare, Narita, Chiba, Japan

The revised manuscript has been updated based on reviewer feedback with the following changes:

1. **Data Analysis Description**: This section has been streamlined to highlight essential steps and findings, removing non-essential technical details to improve clarity. A concise overview now explains how exploratory factor analysis helped identify factors and validate the rubric.
2. **Language and Terminology**: Revisions have made the language more accessible and straightforward, reducing technical jargon and conveying complex concepts.
3. **Curriculum Development, Training, and Patient Care**: The discussion section has been expanded to link the results to implications for curriculum development, training, and patient care, including specific examples to demonstrate the impact of the findings.
4. **Study Limitations**: Detailed elaboration on the study's limitations discusses the limited sample size and its effect on the generalizability of the findings, especially in diverse cultural contexts, with implications for future research directions.
5. **Rubric Creation Process**: The initial rubric creation process has been clarified, noting that the linguistic and clinical communication criteria are already established assessment points used in the OET rubric.
6. **Authorship Criteria**: Although four physicians contributed to the rubric's adaptation, only the primary author met the ICMJE's authorship criteria. Contributions from the other three are acknowledged in the Acknowledgements section.
7. **Timeframe of Rubric Creation**: The revised manuscript includes the timeline of the rubric's development, specifying three months from April to June 2022.
8. **Single Institution Data Limitation**: The manuscript acknowledges the limitations of data derived from a single institution and adjusts the concluding statements accordingly.
9. **Assessment Criteria Terminology**: "Equitable" has been replaced with "strict and objective" to describe the assessment criteria.
10. **Rubric Description and Clarity**: Rubric items are now entirely written in the text to improve clarity.
11. **Rubric Naming Sentence**: The sentence describing the rubric's name has been revised to improve clarity.
12. **AI Tool Acknowledgment**: AI tools like ChatGPT and Grammarly are explicitly acknowledged for proofreading and enhancing manuscript clarity.The revised manuscript has been updated based on reviewer feedback with the following changes:
13. **Data Analysis Description**: This section has been streamlined to highlight essential steps and findings, removing non-essential technical details to improve clarity. A concise overview now explains how exploratory factor analysis helped identify factors and validate the rubric.
14. **Language and Terminology**: Revisions have made the language more accessible and straightforward, reducing technical jargon and conveying complex concepts.
15. **Curriculum Development, Training, and Patient Care**: The discussion section has been expanded to link the results to implications for curriculum development, training, and patient care, including specific examples to demonstrate the impact of the findings.
16. **Study Limitations**: Detailed elaboration on the study's limitations discusses the limited sample size and its effect on the generalizability of the findings, especially in diverse cultural contexts, with implications for future research directions.
17. **Rubric Creation Process**: The initial rubric creation process has been clarified, noting that the linguistic and clinical communication criteria are already established assessment points used in the OET rubric.
18. **Authorship Criteria**: Although four physicians contributed to the rubric's adaptation, only the primary author met the ICMJE's authorship criteria. Contributions from the other three are acknowledged in the Acknowledgements section.
19. **Timeframe of Rubric Creation**: The revised manuscript includes the timeline of the rubric's development, specifying three months from April to June 2022.
20. **Single Institution Data Limitation**: The manuscript acknowledges the limitations of data derived from a single institution and adjusts the concluding statements accordingly.
21. **Assessment Criteria Terminology**: "Equitable" has been replaced with "strict and objective" to describe the assessment criteria.
22. **Rubric Description and Clarity**: Rubric items are now entirely written in the text to improve clarity.
23. **Rubric Naming Sentence**: The sentence describing the rubric's name has been revised to improve clarity.
24. **AI Tool Acknowledgment**: AI tools like ChatGPT and Grammarly are explicitly acknowledged for proofreading and enhancing manuscript clarity.

# Response

**Published:** 2024-08-24

**Authors:** TAKAYUKI OSHIMI

**Affiliations:** School of Medicine, International University of Health and Welfare, Narita, Chiba, Japan

Thank you for your thorough review and insightful comments on my manuscript. I greatly appreciate your time and effort to provide constructive feedback. Below, I have addressed each of your comments in detail.   

**Reviewer’s Comment:**

***Firstly, I feel that while the testing of the rubric is well described, its initial creation process seems to be somewhat vague. In the section “Origins of the assessment framework” it is not clear whether the linguistic criteria and clinical communication criteria are extracted from the OET by the author or already established assessment points (rubric) already used for the OET.***   

**Response:**

 Thank you for your insightful feedback and for bringing attention to the clarity of the rubric's initial creation process. I have made revisions to the manuscript to address your concerns as follows: Drawing from these comprehensive parameters of the OET, the researcher aims to design a robust assessment tool tailored to the needs of EFL medical undergraduates for history-taking skills. The linguistic and clinical communication criteria, which are already established assessment points used in the OET rubric, were adapted with modifications to enhance clarity and objectivity, making them more user-friendly for clinicians involved in the assessment process.   

**Reviewer’s Comment:​​​​​​​**

***Next the process of “adapting a rubric” seems to state that it was done by 4 people. I wonder why they are not listed as authors on the paper if they have created and tested the rubric.***   

**Response:**

 The rubric adaptation process was indeed carried out with contributions from four physicians, including myself. However, the other three physicians do not meet the criteria for authorship as recommended by the International Committee of Medical Journal Editors (ICMJE). According to the ICMJE, authorship should be based on the following four criteria:

- Substantial contributions to the conception or design of the work; or the acquisition, analysis, or interpretation of data for the work; AND
- Drafting the work or reviewing it critically for important intellectual content; AND
- Final approval of the version to be published; AND
- Agreement to be accountable for all aspects of the work in ensuring that questions related to the accuracy or integrity of any part of the work are appropriately investigated and resolved.

While the other three physicians played a significant role in the creation of the rubric, they did not participate in the actions as described above. Therefore, they do not fulfill the authorship criteria. However, I have acknowledged their valuable contributions to the rubric in the **Acknowledgements** section of the paper.   

**Reviewer’s Comment:​​​​​​​**

***No timeframe is given as to when the rubric was made and how long it took to make.*** 

**Response:**

 In response to your comment, I have revised the manuscript to include the timeframe during which the rubric was created. The discussions and development of the rubric were conducted over a period of three months, from April 2022 to June 2022.   

**Reviewer’s Comment:​​​​​​​**

***I think the major limitation of this paper is the fact that the rubric was tested at a single institution in Japan; therefore, it is impossible to state, “In summary, the LCM Rubric Version 1, informed by the OET speaking sub-test criteria, provides a tailored approach for assessing the English history-taking skills of EFL medical undergraduates.” I think you have to temper the statement here a little, and in the limitations section, state clearly that the data are derived from a single institution in Japan, which limits the generalizability of the findings. ***   

**Response:**

 In response to your comments, I have revised the manuscript to acknowledge the limitation that the data are derived from a single institution. I have also adjusted the concluding statement to reflect this consideration. In summary, the LCM Rubric Version 1, informed by the OET speaking sub-test criteria, provides a tailored approach for assessing the English history-taking skills of EFL medical undergraduates. The findings about the rubric's validation and reliability suggest its potential utility in educational settings, although the results should be interpreted with caution due to the study's limitations. They bear implications for curriculum development and student training in global medical communication.

**Limitations:**

 This study has a number of limitations. Firstly, it examines the English history-taking abilities of EFL medical undergraduates at a single private Japanese medical school. The unique emphasis on English at IUHW may limit the findings' generalizability to other academic settings. During the first two years of their medical education, IUHW students were extensively trained in medical subjects taught in English. This foundation was bolstered by a 120-hour medical English course specifically designed to enhance their English history-taking proficiency. Given its primary focus on Japan, it is also crucial to consider linguistic nuances in different international medical contexts.

 Relying only on the OET speaking sub-test might omit some aspects of students' linguistic proficiency. The selection of assessors, despite their diversity, introduces potential bias, and limited data on them could affect the study's depth. The specific focus on migraines and a particular cohort may not capture the broader EFL medical experience. Video recordings, while practical, might only partially capture face-to-face interactions. Concerns about sample representativeness and the influence of access to post-CC OSCE scores on the study’s objectivity are worth noting.   

**Reviewer’s Comment:​​​​​​​**

***What does the author mean by “equitable” in reference to the assessment criteria for EFL medical students? Maybe “stringent and objective criteria” would be a more appropriate?***  

**Response:**

 In response to your comment, I have revised the text as follows: "the objective was to develop strict and objective criteria to assess the English history-taking proficiency of EFL medical undergraduates."   

**Reviewer’s Comment:​​​​​​​**

***The information in following sentence seems to be a repetition of that given in the preceding sentences. “This adaptive approach was made necessary by the elective status of the TOEFL ITP following the third year of their study.”***   

**Response:**

 In response to your comment, I have revised the text as follows: "We evaluated the construct validity of our rubric through exploratory factor analysis (EFA). This analysis included 13 rubric items and 12 TOEFL ITP score variables, reflecting English proficiency at different stages of medical education. Using the Kaiser criterion and Promax rotation, we identified key factors that validate our rubric. It is important to note that due to optional participation in the TOEFL ITP exams after the third year, we occasionally used scores from earlier tests as substitutes to handle missing data effectively."   

**Reviewer’s Comment:​​​​​​​**

***In the Results section the authors often refer to items in the rubric by an alpha-numeric identifier such as in this sentence: “A notable ceiling effect was discerned for eight items: L3, C1, C2, C3, C4, M1, M2, and M3.” This is not very helpful for the reader, as they have to constantly refer to the tables to understand what these identifiers refer to. I think it would be better to write them out in full throughout the text.***   

**Response:**

 In response to your comment, I have revised the manuscript to write out the rubric items in full throughout the text, using the following labels:

- L1 (Intelligibility)
- L2 (Fluency)
- L3 (Appropriateness of Language)
- L4 (Resources of Grammar and Expression)
- C1 (Relationship Building)
- C2 (Understanding & Incorporating the Patient's Perspective)
- C3 (Providing Structure)
- C4 (Information Gathering)
- M1 (Patient Safety)
- M2 (Medical History Components)
- M3 (Comprehensiveness of History of Present Illness)
- G1 (Global Rating 1)
- G2 (Global Rating 2)

However, in one section, I opted for a different style to enhance readability: "The three factors identified in the EFA are described as follows: Factor 1, labeled 'Linguistic-Clinical Distinction,' differentiates between linguistic aspects (L) and clinical components (C & M). Factor 2, labeled 'Communication Dynamics,' encompasses elements that foster doctor-patient rapport-building (G1), facilitate information gathering (C4), and include other relevant linguistic and medical tasks (L & M). Thirdly, Factor 3, labeled 'Medical Comprehension,' highlights the subtleties in medical history components (M2), the comprehensiveness of the history of the present illness (M3), and the interpretation of patient perspectives (C2)." I believe this approach keeps a balance between clarity and readability, making the section more accessible to readers while maintaining the integrity of the analysis. 

**Reviewer’s Comment:​​​​​​​**

***This sentence sounds unnatural: “The adapted rubric focused on history-taking is termed the LCM Rubric Version 1.” How about something like “The adapted rubric was named the LCM Rubric Version 1, with the acronym "LCM" denoting the three pillars of the rubric: Linguistic, Clinical Communication, and Medical Interview.” ***   

**Response:**

 In response to your comment, I have revised the sentence as instructed to: "The adapted rubric was named the LCM Rubric Version 1, with the acronym 'LCM' denoting the three pillars of the rubric: Linguistic, Clinical Communication, and Medical Interview."

**Reviewer’s Comment:​​​​​​​**

***The English grammar here is confusing: “The study examining English history-taking abilities of EFL medical undergraduates at a private Japanese medical school has limitations.” Something like this would be better: “This study has a number of limitations. Firstly…”.*** ***You state the following: “The unique emphasis on English at IUHW may limit the findings' generalizability to other academic settings.” You might need to clarify the “unique emphasis on English at IUHW” for the reader by comparing it to the English language training at a typical Japanese medical school curriculum.***   

**Response:**

 In response to your comments, I have revised the section to improve clarity and provide a more detailed explanation of the unique emphasis on English at IUHW. 

**Limitations:**

 This study has a number of limitations. Firstly, it examines the English history-taking abilities of EFL medical undergraduates at a single private Japanese medical school. The unique emphasis on English at IUHW may limit the findings' generalizability to other academic settings. During the first two years of their medical education, IUHW students were extensively trained in medical subjects taught in English. This foundation was bolstered by a 120-hour medical English course specifically designed to enhance their English history-taking proficiency. Given its primary focus on Japan, it is also crucial to consider linguistic nuances in different international medical contexts.

 Relying only on the OET speaking sub-test might omit some aspects of students' linguistic proficiency. The selection of assessors, despite their diversity, introduces potential bias, and limited data on them could affect the study's depth. The specific focus on migraines and a particular cohort may not capture the broader EFL medical experience. Video recordings, while practical, might only partially capture face-to-face interactions. Concerns about sample representativeness and the influence of access to post-CC OSCE scores on the study’s objectivity are worth noting.******

**Reviewer’s Comment:​​​​​​​**

***I think the following sentence is AI generated: “This study underscores the importance of expansive inter-institutional collaboration between medical schools in Japan and their international counterparts to achieve a richer, more diverse participant base.” In light of my comment above, I think it would be better to say something like, for example: “To further investigate the applicability of the rubric to contexts beyond that of the single institution featured in the current study, further research involving other institutes in Japan and overseas is necessary.”***   

**Response:**

 In response to your comment, I have revised the sentence as follows: "Further research involving other institutes in Japan and overseas is necessary to investigate the rubric's applicability to contexts beyond that of the single institution featured in the current study. Integrating artificial intelligence with the current rubric could optimize assessments in terms of efficiency and cost. Feedback from assessors on the LCM Rubric Version 1 will further inform assessment refinement. Although this research provides valuable insights into the English history-taking abilities of EFL medical undergraduates at IUHW, the highlighted limitations set the direction for future studies."  The use of AI is addressed in response to the following comment.  

**Reviewer’s Comment:​​​​​​​**

***This is a stylistic point, but in the examples below, you give agency to things that are not alive: “This study aimed to create…” “This research endeavors to…”  “this research aims to devise…” “the present research aims to design…”*** ***The researcher has the human agency to create, devise, endeavor, design, while a study or research cannot. So, it is better to express this something like: “The aim of the study was…” “The purpose of this research was…”***   

**Response:**

 In response to your comment, I have revised the relevant sections as instructed, using phrasing such as "The aim of the study was..."  to better align with your recommendation. 

**Reviewer’s Comment:​​​​​​​**

***Finally, I suspect that ChatGPT has been used in the creation of this manuscript. If so, it should be acknowledged in the manuscript. I also suggest a native English-speaker check.***

**Response:**

 Thank you for your thorough review and concern regarding the use of AI tools in creating this manuscript. I want to clarify that the author wrote the draft, and it was not generated or created by ChatGPT. The author drafted the manuscript, and a qualified English teacher with extensive experience in journal editing carefully edited the draft to enhance clarity and coherence. The author used ChatGPT and Grammarly to assist with proofreading and further enhance the manuscript's clarity. This use of AI tools has been transparently acknowledged in the Acknowledgments section as follows: "The author acknowledges the use of ChatGPT, developed by OpenAI, and Grammarly, developed by Grammarly Inc., to assist with proofreading and enhance the manuscript's clarity." Thank you once again for your valuable feedback and for helping to improve the quality of this manuscript.

# Reviewer report: Reviewer response for version 1

**DOI:** 10.21956/mep.21327.r36689

**Published:** 2024-05-14

**Authors:** Alexandra Lopez Vera

**Affiliations:** 1 California University of Science and Medicine, Colton, California, USA

The article presents the development and validation of the LCM Rubric Version 1, aimed at assessing English history-taking skills among EFL medical undergraduates. It highlights the significance of effective communication in medical practice and the challenges faced by EFL students in English-speaking healthcare environments. Drawing inspiration from the OET speaking sub-test, the rubric is designed to evaluate linguistic and clinical communication criteria relevant to history-taking tasks. The study utilizes a comprehensive methodology, including ethical considerations, participant consent, and data analysis techniques such as exploratory factor analysis and multiple regression analysis. Findings indicate promising construct validity and reliability of the rubric, with key criteria showing significant associations with clinical competency ratings. Limitations are acknowledged, including the study's focus on a single institution and potential biases in assessor selection. Recommendations for further research include expanding collaboration between institutions and integrating artificial intelligence into assessment processes.

 1. The description of the data analysis process is overly detailed, potentially leading to confusion. I recommend streamlining the description by focusing on essential steps and findings while omitting less relevant technical aspects. Ensure logical flow and clarity throughout. For example, instead of delving into technical details, focus on summarizing the main findings of the data analysis process. For example, rather than describing each step of exploratory factor analysis, provide a concise overview of how factors were identified and their implications for the validation of the rubric (Oshimi, 2024).

 2. Through the article, the language seems robotic. Simplify language and terminology, particularly in sections discussing statistical analyses and methodology, to improve readability and accessibility. Use clear and concise language to convey complex concepts, avoiding unnecessary jargon or technical terms.

 3. Discuss how the results could inform curriculum development, student training, and patient care outcomes, offering specific examples or scenarios.  For instance, "Our findings suggest that certain criteria, such as the comprehensiveness of the history of present illness (M3), significantly contribute to clinical competency. This underscores the importance of prioritizing comprehensive training in medical history-taking skills for EFL medical students to enhance their clinical practice."

 4. Elaborate on the potential impact of limitations on study outcomes. Instead of a vague statement, provide specific examples. For instance, "The limited sample size might affect the generalizability of our findings, particularly in diverse cultural contexts. Future research should aim to replicate our study with larger and more diverse participant samples to confirm the robustness of our findings."

Have any limitations of the research been acknowledged?

Partly

Is the work clearly and accurately presented and does it cite the current literature?

Yes

If applicable, is the statistical analysis and its interpretation appropriate?

Partly

Are all the source data underlying the results available to ensure full reproducibility?

Yes

Is the study design appropriate and is the work technically sound?

Yes

Are the conclusions drawn adequately supported by the results?

Yes

Are sufficient details of methods and analysis provided to allow replication by others?

Yes

Reviewer Expertise:

Minority Health, Medical Education, Medical Spanish

I confirm that I have read this submission and believe that I have an appropriate level of expertise to confirm that it is of an acceptable scientific standard, however I have significant reservations, as outlined above.

# Response

**Published:** 2024-08-03

**Authors:** TAKAYUKI OSHIMI

**Affiliations:** School of Medicine, International University of Health and Welfare, Narita, Chiba, Japan

1. **Reviewer's comment:**
** Streamlining the Data Analysis Description:**
**Response:** Thank you for your constructive feedback on the data analysis section. I have streamlined this section to focus on the essential steps and findings, omitting less relevant technical details to enhance clarity. The descriptions have been revised to provide a concise overview of how factors were identified through exploratory factor analysis and their implications for validating the rubric. These changes aim to simplify the presentation and improve the logical flow of the information.
2. **Reviewer's comment:**
** Simplification of Language and Terminology:**
**Response:** I appreciate your comments regarding the language and terminology used in my manuscript. In response, I have revised the language throughout the article to make it more accessible and less robotic. Technical jargon has been minimized, and complex concepts are conveyed in clear, concise language. This revision should make the statistical analyses and methodology sections more accessible to read and understand.
3. **Reviewer's comment:**
** Implications for Curriculum Development, Training, and Patient Care:**
**Response:** I have expanded the discussion section to clearly articulate how the results can inform curriculum development, student training, and patient care outcomes. Specific examples have been added to illustrate the impact of my findings, such as the importance of comprehensive training in medical history-taking skills for EFL medical students, which is crucial for enhancing clinical practice. These additions should provide more explicit guidance on the practical applications of my research.
4. **Reviewer's comment:**
** Elaboration on the Impact of Study Limitations:**
** Response:** In response to your suggestion, I have elaborated on the potential impacts of the study's limitations. I now include specific examples, such as the limited sample size and its effect on the generalizability of my findings, particularly in diverse cultural contexts. I also discuss the implications of these limitations for future research directions, emphasizing the need for replication studies with more extensive and varied participant samples to confirm the robustness of my findings. I hope these revisions address your concerns and enhance the manuscript's clarity and impact. Thank you for your guidance and the opportunity to improve my work.
